# The Composition of Volatiles and the Role of Non-Traditional LOX on Target Metabolites in Virgin Olive Oil from Autochthonous Dalmatian Cultivars

**DOI:** 10.3390/molecules29081696

**Published:** 2024-04-09

**Authors:** Barbara Soldo, Maja Jukić Špika, Igor Pasković, Elma Vuko, Marija Polić Pasković, Ivica Ljubenkov

**Affiliations:** 1 Department of Chemistry, Faculty of Science, University of Split, Ruđera Boškovića 33, 21000 Split, Croatia; barbara@pmfst.hr; 2 Department of Applied Sciences, Institute for Adriatic Crops and Karst Reclamation, Put Duilova 11, 21000 Split, Croatia; maja@krs.hr; 3 Centre of Excellence for Biodiversity and Molecular Plant Breeding, Svetošimunska 25, 10000 Zagreb, Croatia; 4 Department of Agriculture and Nutrition, Institute of Agriculture and Tourism, K. Huguesa 8, 52440 Poreč, Croatia; paskovic@iptpo.hr (I.P.); mpolic@iptpo.hr (M.P.P.); 5 Faculty of Health Studies, University of Rijeka, Viktora Cara Emina 5, HR-51000 Rijeka, Croatia; 6 Department of Biology, Faculty of Science, University of Split, Ruđera Boškovića 33, 21000 Split, Croatia; elma@pmfst.hr

**Keywords:** lipoxygenase enzyme, virgin olive oil, maturity index, harvest time, 9- and 13-hydroperoxides of octadecenoic acid, volatile compounds, total phenolic compounds, Oblica, Levantinka and Lastovka cultivars

## Abstract

The lipoxygenase pathway has a significant influence on the composition of the volatile components of virgin olive oil (VOO). In this work, the influence of the maturity index (MI) on the activity of the lipoxygenase enzyme (LOX) in the fruits of the autochthonous Dalmatian olive cultivars Oblica, Levantinka and Lastovka was studied. The analysis of the primary oxidation products of linoleic acid in the studied cultivars showed that LOX synthesises a mixture of 9- and 13-hydroperoxides of octadecenoic acid in a ratio of about 1:2, which makes it a non-traditional plant LOX. By processing the fruits of MI~3, we obtained VOOs with the highest concentration of desirable C6 volatile compounds among the cultivars studied. We confirmed a positive correlation between MI, the enzyme activity LOX and the concentration of hexyl acetate and hexanol in cultivars Oblica and Lastovka, while no positive correlation with hexanol was observed in the cultivar Levantinka. A significant negative correlation was found between total phenolic compounds in VOO and LOX enzyme activity, followed by an increase in the MI of fruits. This article contributes to the selection of the optimal harvest time for the production of VOOs with the desired aromatic properties and to the knowledge of the varietal characteristics of VOOs.

## 1. Introduction

Virgin olive oil (VOO), a food with high nutritional value and unique sensory properties, is associated with the Mediterranean diet, whose benefits for human health have been described in several scientific papers [1,2,3]. Among the positive sensory properties of VOO, besides pungency and bitterness, is fruitiness, which is described as the set of aromatic properties that characterise the oil obtained from healthy and fresh, green or ripe fruit, depending on the cultivar, and observation can be direct or indirect, i.e., retronasal. The unique odour of VOO is the result of a complex mixture of volatile compounds formed during and after the extraction of the oil from the olive fruit [4,5,6,7]. Most of these volatile substances belong to the aldehydes, alcohols, esters, hydrocarbons, ketones, and furans, with the C6 and C5 volatile compounds being the most common. The analysis of volatiles in the VOOs of different Italian and Spanish olive cultivars revealed that C6 aldehydes (hexanal, *Z*-3-hexenal, *E*-2-hexenal), C6 alcohols (hexanol, *Z*-3-hexenol, *E*-2-hexenol) and their acetate esters (hexyl acetate and Z-3-hexenyl acetate) account for 60% to 80% of the total volatiles [8]. Previous studies on the aroma of VOOs indicate the dominance of C6 volatiles, among which *E*-2-hexenal stands out. Volatiles with desirable odour properties belong to specialised plant metabolites and are formed in a series of biochemical reactions known as the lipoxygenase (LOX) pathway. The enzymes of the LOX pathway are released during processing after the tissue of the olive fruit has been ruptured. Precursors of the volatile C6 compounds are linoleic acid (18:2) and linolenic acid (18:3) [8]. The corresponding hydroperoxides of these acids are formed by the action of the enzyme LOX. To date, four LOX enzyme isoforms have been isolated from olive fruits, two representatives each of the LOX-1 and LOX-2 superfamilies. The isoforms of the LOX-1 superfamily show a higher affinity for linoleic acid and synthesise 9- and 13-*Z*,*E*-HPOD in a ratio of 2:1 and 4:1, respectively, whereas the representatives of the LOX-2 superfamily show a higher affinity for linolenic acid and synthesise exclusively 13-hydroperoxides of fatty acids [9,10,11]. Hydroperoxides of fatty acids are cleaved by hydroperoxide lyase (HPL), and the resulting aldehydes are reduced to alcohols by alcohol dehydrogenase (ADH) and converted to the corresponding esters by acyltransferase (ATT) [5,12]. In addition to this main action, the enzyme LOX can also cleave the resulting hydroperoxides of fatty acids, and the released alkoxy radicals participate in chemical reactions, whose products are volatile C5 compounds, of which only 1-penten-3-one contribute independently to the desirable odour characteristics of VOO, while the other C5 compounds contribute to the aroma through a synergistic effect with other volatile compounds [13]. Ultimately, the content of volatile compounds in VOO strongly depends on the cultivar, the ripeness of the fruit, the geographical area of cultivation, the agronomic factors, and the processing method [14,15,16,17].

There are around 40 autochthonous olive cultivars in Croatia, most of which are cultivated in extensive olive groves. The Oblica variety accounts for 60% of the total Croatian olive assortment and is the most cultivated variety in Dalmatia. Lastovka is another important autochthonous cultivar grown in central and southern Dalmatia and on the island of Korčula. In terms of the number of parent trees, the Levantinka cultivar is in third place with the largest population on the island of Šolta. Although the content of volatile compounds in VOO has been actively researched for three decades, there is very little data in the literature on the presence of volatiles, especially during fruit ripening, for the studied cultivars [18,19].

Therefore, the first objective of this work was to study the specific activity of the enzyme LOX in the cultivars Oblica, Levantinka and Lastovka when its substrate is linoleic acid, focusing the research on the study of a branch of the LOX pathway starting from 13-HPOD and targeting volatile saturated compounds with hexanal, hexanol and hexyl acetate. Since the fruitiness is derived from the total content of volatile C6 compounds in the VOO, the second objective of the work is to determine the content of other volatile compounds in the oils of the crops studied. The biochemical changes that take place during ripening are also reflected in the quantity and composition of phenolic compounds (PC) in the fruit. PCs are the primary antioxidants that maintain the stability of the VOO and are carriers of bitterness and pungency and, together with the volatiles, influence flavour and aroma [20,21]. Although the proportion of total phenolic compounds (TPCs) in olive oil mostly depends on the cultivar [22,23], several studies confirm that oils obtained by processing low-ripeness fruits contain a higher proportion of these compounds [21,24,25]. Due to the known inhibitory effect of PC on the enzyme LOX [26], one of the objectives of this work was to observe the change in TPC concentration during fruit ripening. The results obtained form the basis for determining the optimum harvest time (degree of ripeness of the fruit) in order to obtain monocultivar oils with the highest possible content of desired volatile compounds in the subsequent production process. The analysis of LOX enzyme activity, TPC content and volatile components during the ripening period of the olive fruit can lead to new insights into the development of the aroma and properties of oils from cultivars that have been little studied to date.

## 2. Results and Discussion

### 2.1. Harvest Times and Fruit Maturity Index

In order to achieve the objectives set, the olive fruits were harvested in three trial years during the ripening period from the beginning of October to mid-December. Five to six harvest times (HTs) were carried out per year, each two weeks apart. The fruit maturity index (MI) was determined at each HT according to the method presented by Uceda and Frias [27]. The values of the fruit MI for the cultivars Oblica, Levantinka and Lastovka in the period from 2013 to 2015 are shown in Table 1.

The cultivars differed in terms of MI values at the same harvest time, which is an expected cultivar trait. Oblica and Levantinka belong to the early cultivars. The MI values of Oblica fruits ranged from 0.23 to 4.98 (Table 1), while those of Levantinka ranged from 0.43 to a high value of 5.88. Lastovka had the highest initial MI value of about 0.8 in all study years, and during maturation, the least changes in MI values were observed, with a maximum of 3.64. The slower growth of MI is one of the reasons why Lastovka is classified as a medium–early cultivar.

### 2.2. Lipoxygenase Activity in Olive Fruits

**Figure 1:**
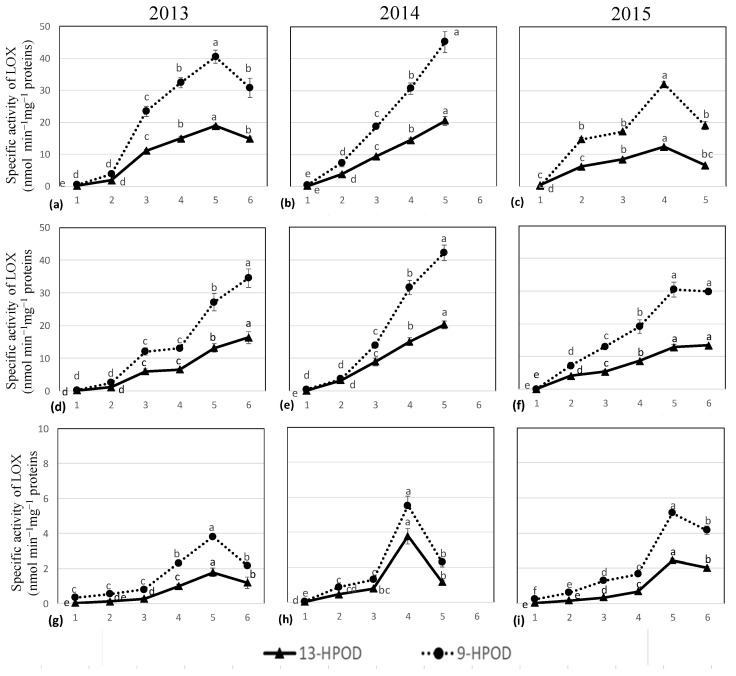
The figure shows the results of the specific activity of lipoxygenase (LOX) (nmol min^−1^ mg^−1^ proteins) in the cultivars Oblica (**a**–**c**), Levantinka (**d**–**f**) and Lastovka (**g**–**i**) depending on the harvest time (1–6) and the year of study (2013, 2014 and 2015). The products of specific enzyme activity are 9-hydroperoxylinolic acid (9-HPOD) and 13-hydroperoxylinolic acid (13-HPOD). The results represent the mean of three determinations of specific activity ± SD. Different lowercase letters linked to each enzyme activity product represent statistically significant differences between 13-HPOD and 9-HPOD mean values at different harvest times (for the specific study year and cultivar) obtained via a one-way ANOVA and Tukey’s post hoc test (at *p* ≤ 0.05).

Plant LOX enzymes differ in their positional specificity, and as far as the oxygenation of linoleic acid is concerned, they are divided into 9-LOX and 13-LOX. The results of the HPLC analysis showed that the enzyme LOX isolated from the fruits of Oblica, Levantinka and Lastovka during the ripening period had a double position specificity (Figure 1).

The product specificity results of the enzyme LOX showed a mixture of 9- and 13-hydroperoxides in a ratio of about 2:1, which classified this enzyme as a non-traditional plant LOX enzyme (Figure 1) [9,28]. In general, as ripening progressed, a significant increase in the specific activity of the LOX enzyme was observed in the fruits of all three cultivars (Figure 1a–i, Supplementary Materials, Table S4). In the first two years, the specific activity values in the Oblica fruits were approximately the same, and the enzyme reached the highest activity at 5 HT in 2013 (40.44 ± 2.05 nmol 9-*Z*,*E*-HPOD min−^1^ mg^−1^ proteins and 18.90 ± 0.55 nmol 13-*Z*,*E*-HPOD min^−1^ mg^−1^ proteins), and 2014 (45.23 ± 3.26 nmol 9-*Z*,*E*-HPOD min−^1^ mg^−1^ proteins and 20.58 ± 1.39 nmol 13-*Z*,*E*-HPOD min^−1^ mg^−1^ proteins). In the third year of the study, a slightly lower maximum value was reached at 4 HT (Figure 1a–c). A similar trend with increasing LOX enzyme activity was observed in Levantinka fruits (Figure 1d–f). However, in this cultivar, the highest enzyme activity was measured at the last HT in all study years, with the maximum value in 2014 (42.21 ± 2.39 nmol 9-*Z*,*E*-HPOD min^−1^ mg^−1^ proteins and 20.24 ± 1.14 nmol 13-*Z*,*E*-HPOD min^−1^ mg^−1^ proteins). In general, the values of the specific activity of the LOX enzyme were similar in the Oblica and Levantinka cultivars in the first two years, while slightly lower values were recorded in the third year of the study. On the other hand, lower values for the specific activity of the LOX enzyme were recorded for the Lastovka cultivar in all three years (Figure 1g–i). The trend of increasing enzyme activity can also be observed here, and the highest values were recorded each year in the penultimate harvest periods. The highest values of the specific activity of the LOX enzyme in Lastovka fruits were 5.52 ± 0.54 nmol 9-*Z*,*E*-HPOD min^−1^ mg^−1^ proteins and 3.79 ± 0.44 nmol 13-*Z*,*E*-HPOD min^−1^ mg^−1^ proteins and were measured in 2014. The few studies that have monitored the activity of the LOX enzyme during fruit development and ripening have so far produced contradictory results. Two teams reported the highest LOX activity in green fruits at week 17 after pollination [29] and earlier at week 13 after pollination [30], respectively. Palmieri-Thiers et al. (2009) studied the change in LOX activity during fruit development from small green to fully ripened black olives. Since they conducted their studies on three Italian and one Spanish cultivars, they also showed that the activity of the enzyme LOX is influenced by the cultivar. Moreover, they observed an increase in the enzyme activity of LOX during ripening in all cultivars, especially during the period when the colour of the fruit skin changes from green to purple, with the highest enzyme activity observed in dark, fully ripe fruits [31]. The presence of different LOX isoforms in olive fruits during their development could explain these contradictory results. Molecular studies confirmed the presence of four LOX-isoenzymes in olive tissues, two of which (one each from the LOX-1 and LOX-2 superfamilies’) show intense expression during olive ripening [10,11], while the other two are more abundant in green fruits [32]. The role of the LOX enzyme in olives is not yet fully understood, nor is the possible influence of some environmental factors (e.g., temperature and rainfall) on enzyme activity. By determining the pattern of LOX gene expression during fruit development as well as their regional specificity, it would be possible to learn more about the contribution of each isoform to the biosynthesis of VOO flavour.

### 2.3. Olive Oil Qualitative Parameters

The results of the physicochemical analysis of the oils of Oblica, Levantinka, and Lastovka cultivars are listed in Supplementary Materials, Tables S1–S3. The percentage of FFA ranged from 0.19 to 0.65% oleic acid in the oils of the Oblica cultivar, from 0.15 to 0.40% oleic acid in the oils of the Levantinka cultivar, and from 0.18 to 0.79% oleic acid in the oils of the Lastovka cultivar, which means that these oils can be classified as extra virgin olive oils (EVOOs). The only exception to EVOO is Oblica oil, which was last harvested in 2013. Due to its FFA content of 1.51%, this oil is classified in a lower category, VOO. The three-year peroxide value (PV) values ranged from 3.94 to 16.02 for Oblica oils, from 4.22 to 14.72 for Levantinka oils, and from 3.84 to 9.18 meq O_2_ kg^−1^ for Lastovka oils. The results of the measurement of specific absorbance in the UV range (K_232_ and K_270_) classify the oils as EVOOs (Supplementary Materials, Tables S1–S3). All the results obtained were compared with the limits established by the international regulations EU Regulation 2022/2104 [33] and classified as above. The values obtained (Supplementary Materials, Tables S1–S3) are consistent with previous studies on monovarietal oils, which show that these parameters are not cultivar-dependent and that there are no differences between monovarietal oils [34,35]. Although there is no clear correlation between the basic quality parameters and the MI of the fruit [36], the fruits of olives with a higher degree of MI are softer and, therefore, more susceptible to hydrolytic and oxidative changes, which could be reflected in the quality changes observed in Oblica oils from overripe olive fruits from 2013. During the ripening of olives, the composition of the oil changes, but these changes are not the same for all cultivars [37].

### 2.4. Influence of Cultivar, Harvest Time and Enzyme Activity LOX on the Amount of Total Phenolic Compounds in Virgin Olive Oils 

**Figure 2:**
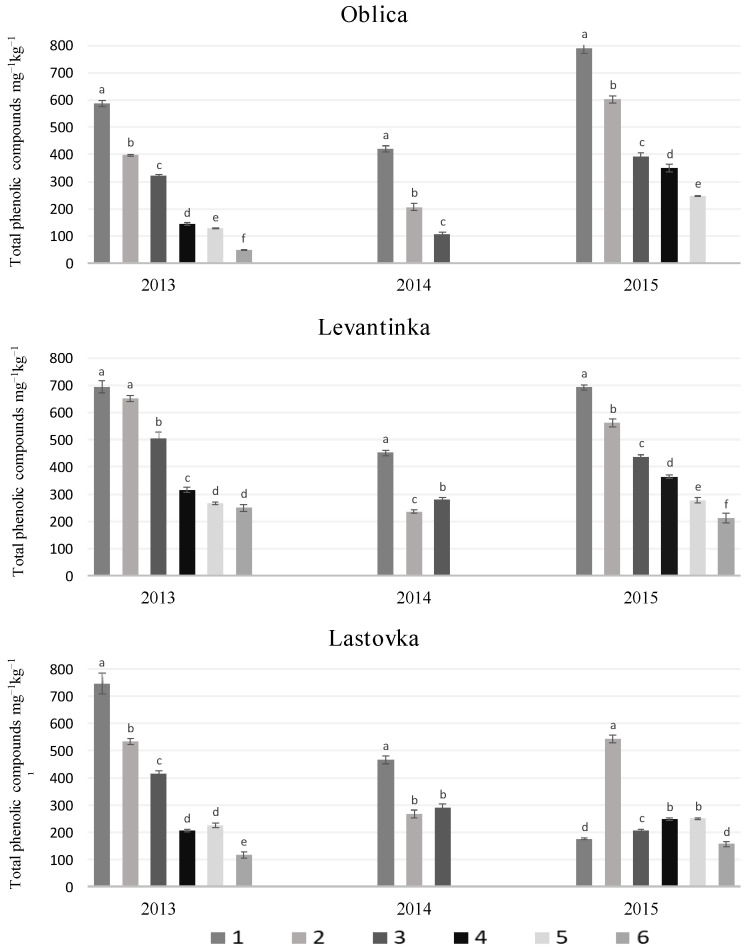
The figure shows the concentration of total phenolic compounds (TPCs) (mg kg^−1^) in VOO from cultivars Oblica, Levantinka and Lastovka as a function of harvest time (1–6) and year of study (2013, 2014 and 2015). The results represent the mean of three determinations ± SD. Different lowercase letters represent statistically significant differences between TPC mean values at different harvest times (for the specific study year and cultivar) obtained via a one-way ANOVA and Tukey’s post hoc test (at *p* ≤ 0.05).

Phenolic compounds are the most important antioxidants; they ensure the oxidative stability of the oil and, together with volatile components, influence flavour and aroma [38,39,40]. Various phenolic compounds, including flavonoids (e.g., apigenin and luteolin), phenolic alcohols (e.g., tyrosol and hydroxytyrosol), and phenolic acids (e.g., caffeic acid and vanillic acid), alongside a predominant group of secoiridoids, have been isolated from virgin olive oil, with the dialdehyde forms of decarboxymethyloleuropein aglycone and decarboxymethyl ligstroside aglycone emerging as the most prevalent. [41,42]. The values of total phenolic compounds (TPCs) in Oblica VOO ranged from 50.5 to 790.7 mg kg^−1^, in Levantinka from 212.3 to 694.1 mg kg^−1^, and in Lastovka from 116.2 to 745.7 mg kg^−1^ (Figure 2, Supplementary Materials, Table S4).

In all three years, we observed an almost linear decrease in the TPC content in VOOs of the studied cultivars with increasing fruit maturity (Figure 2). Correlation analysis revealed a strong negative correlation between TPC and maturity index parameter (MI) (R = 0.619, *p* < 0.001 for Oblica; R = 0.751, *p* < 0.001 for Levantinka and R = 0.679, *p* < 0.001 for Lastovka cultivar, respectively. An exception to this trend was observed in the VOOs of the cultivar Lastovka from 2015. Almost all VOOs contained an almost equal amount of TPCs (~200 mg kg^−1^), while VOO 2 HT stood out with an almost threefold higher TPC content. In general, lower TPC contents were found in the analyzed VOOs of all three cultivars in 2014. Considering the average monthly rainfall, 2014 was characterised by higher average rainfall in July (five times) and September (three times) than in 2013 and 2015 (Supplementary Materials, Figure S1). Results from previous studies on the influence of irrigation indicate lower TPC levels in olive fruits exposed to a greater amount of water [43,44]. It is possible that fruit infestation contributed to the lower TPC levels in 2014. This result was recently confirmed by Notario et al. (2022). The suggestion that there is a stronger oxidative degradation of PC was confirmed by the discovery of a strong induction of the newly discovered polyphenol oxidase in such oils [45]. The results obtained in this study for TPC content are within the wide range of values previously reported for this cultivar characteristic [46,47,48]. A more detailed study of PC composition was carried out on the Oblica and Leccino cultivars depending on the time of harvest and the year of the study. The results indicated lower contents of TPCs, phenolic alcohols and secoiridoids in the oils obtained from ripe fruits [42]. A decrease in TPC content with increasing maturity has also been observed in other cultivars [26,49,50,51]. The explanation for the lower TPC content in VOO can be found in the biochemical changes that occur during ripening. β-glucosidase catalyzes the first step in the synthesis of phenylpropanoid compounds, which are PC precursors. The activity of β-glucosidase is influenced by the degree of fruit ripening and depends on climatic conditions, while on the other hand, stress factors increase the activity of this enzyme [52]. Based on the already known inhibitory effect of phenolic compounds on enzymes [53], including the LOX enzyme [29], its behavioural pattern during ripening was observed. Figure 2 shows the concentration of TPCs in VOOs for the cultivars Oblica, Levantinka and Lastovka as a function of harvest time and year.

Pearson’s linear correlation matrix was used to determine the extent to which these two parameters are related (Figure 1 and Figure 2). As the fruit ripens, an increase in the specific activity of the enzyme LOX and a decrease in the concentration of TPCs in the VOOs is observed, i.e., a significant negative correlation was found between the two parameters mentioned (R = −0.442). Muzzalupo et al. (2012) also reported an inconsistent relationship between the amount of the transcription product of the LOX gene and the TPC content during the ripening process of Coratina fruit [54]. The results of our study and previous studies [21,55] on the influence of PC as a “guardian of oxidative stability” on the synthesis of volatiles in VOO indicate its inhibitory effect on the enzyme LOX. An experiment with the Istrian cultivar Buža, in which PC was added directly to the olive paste during malaxation, resulted in a significantly lower content of volatile C6 aldehydes in the oil [56]. Consequently, the intervention of PC in the LOX metabolic pathway had a cascading effect on enzyme activity. Due to the primary inhibitory effect of PC on the enzyme LOX, a lower amount of 13-hydroperoxyde fatty acids is released into the olive pulp. The substrate deficiency leads to a lower activity of hydroperoxide lyase (HPL), which is also reflected in the activity of alcohol dehydrogenase (ADH), and the slowing down of the main branch of the LOX pathway stimulates an increased synthesis of C5 volatiles. Since we have three years of results, the research carried out represents an important contribution to the understanding of the relationship between the activity of LOX enzymes and the proportion of TPC, with the increase in the MI of fruits. However, the observed increase in enzyme activity (Figure 1) cannot be attributed exclusively to the decrease in TPC content as ripening progresses (Figure 2), as it is also due to the increased expression of two LOX isoforms during this period [11].

### 2.5. Influence of Cultivar, Harvest Time and LOX Enzyme Activity on the Composition of Volatiles in Virgin Olive Oils

Since only the 13-*Z*,*E*-HPOD of linoleic acid is involved in the LOX pathway, the volatile C6 target compounds are hexanal, hexanol and hexyl acetate. In this study, the focus was on observing the change in their concentration in the monocultivar VOOs during fruit ripening (Table 2, Table 3 and Table 4) and the observed differences in concentration related to the activity of the LOX enzyme in olive fruit.

To obtain a more complete picture of the content of desirable C6 and C5 volatile compounds, we determined their concentration in the VOOs of the studied cultivars as a function of MI in all three years of the study. The concentrations of the selected representatives (target compounds) of volatiles from the LOX pathway identified in the VOOs of Oblica, Levantinka and Lastovka, depending on the harvest time and year of study, are presented in Table 2, Table 3 and Table 4, Supplementary Materials, Table S4. As expected, aldehydes (*E*-2-hexenal and hexanal) and alcohols (Z-3-hexen-1-ol, *E*-2-hexen-1-ol and hexanol) dominate among the C6 volatile compounds, while the most common C5 volatile compound is 1-penten-3-ol. The studies conducted so far on the monocultivar VOOs from Dalmatia and Istria have confirmed a high content of C6 volatile aldehydes, especially *E*-2-hexenal [18,34,56,57], which is consistent with the dominance of this compound in other monocultivar VOOs reported worldwide [58,59,60,61,62]. Significant differences in *E*-2-hexenal concentration were observed in all three cultivars depending on the harvest date and the year of the study, but it can be concluded that the richest oils were obtained when processing low to medium fruits fruit (MI = 1.6–3.6) (Table 2, Table 3 and Table 4).

The results obtained are consistent with the pattern previously described for sources of E-2-hexenal [5,62,63,64]. Aroma research has been conducted on numerous cultivars and has revealed different development patterns of E-2-hexenal. A small number of researchers have found higher concentrations of E-2-hexenal in the oils of ripe fruit [65,66]. The second part of the studies suggests that for the desired green odour in VOO, for which this unsaturated aldehyde is best suited, fruits with a lower degree of maturity (MI = 1.5–2) should be used [21,67]. Although these authors did not test the activity of the enzyme LOX, they relate the lower content of C6 aldehyde to the reduction in LOX activity in the later stages of ripening, referring to the work of Salas [68]. In contrast, in this work we monitored the activity of the LOX enzyme during ripening (Figure 1) and analysed the content of C6 volatile compounds in the corresponding VOOs (Table 2, Table 3 and Table 4). Knowing the LOX pathway, we can indirectly relate the specific activity of the LOX enzyme to the concentration of hexanal, the first volatile aldehyde from 13-*Z*,*E*-HPOD, since linoleic acid was used as a substrate.

The maximum hexanal concentration was twice as high in Oblica VOOs (4.49 ± 0.24 mg kg^−1^) (Table 2) as in Lastovka VOOs (2.02 ± 0.13 mg kg^−1^) (Table 4). Levantinka VOOs lie between these two cultivars, with the highest observed hexanal concentration of 3.63 ± 0.31 mg kg^−1^ (Table 3). Considering the results of a three-year study, we conclude that the distribution of hexanal in VOOs from Oblica, Lastovka, and Levantinka cultivars follows the previously described trend of an increase in C6 aldehydes up to the maximum concentration, followed by a decrease in oils from overripe fruits [62,63]. We observed a high content of hexanal in VOO obtained by processing fruits at the mid-maturity stage (MI = 2.07–3.5). Contrary to the described trend, hexanal was only strongly represented in the VOO obtained from green fruits in the Levantinka cultivar in 2015 (MI = 0.68–1.56). It has already been mentioned that the specific activity of the LOX enzyme reached its maximum value in fruits with a purple epidermis colour (MI~4) (R = 0.657), while higher hexanal concentrations were observed in VOO from processed fruits in which half of the fruits had a red colour (MI~3) (Table 2). Almost the same changes in hexanal concentration as a function of harvest time were observed in the Croatian cultivar Buža [57] and the Italian cultivar Coratina [69]. Our results are in agreement with the results of the analysis of the amount of the transcription product of the LOX gene carried out by Muzzalupo et al. (2012), which indicates a continuous accumulation during fruit ripening. In the above-mentioned exception with Levantinka in 2015, the opposite trend was observed for hexanal. It is obvious that we cannot attribute this result to the activity of the LOX enzyme alone. Volatile C6 aldehydes are formed by the action of HPL on the available 13-hydroxyperoxides of fatty acids. We have shown that the activity of the LOX enzyme is low in green fruits (Figure 1), which would imply that there is not enough substrate available for HPL, suggesting that this aldehyde can also be formed via a secondary pathway, i.e., the spontaneous oxidation of fatty acids [5].

Depending on the cultivar and the year of study, the concentration of volatile alcohols varied depending on the time of harvest (Table 2, Table 3 and Table 4). Different patterns of C6 alcohol development have been published in the past, largely depending on the genetic characteristics of the cultivar [65]. The most abundant volatile alcohols in the VOOs of the studied central Dalmatia cultivars are *E*-2-hexen-1-ol and *Z*-3-hexen-1-ol (Table 2, Table 3 and Table 4). The highest concentrations of these alcohols were found in oils obtained by processing ripe fruits (R = 0.243). A higher concentration of *Z*-3-hexen-1-ol compared to *E*-2-hexen-1-ol was found in oils from green fruits of Oblica and Levantinka, and the ratios converge and even shift in favour of *E*-2-hexen-1-ol only in oils from ripe fruits (Table 2 and Table 3). The described trend is also maintained in the Lastovka cultivar, although lower alcohol concentrations were found here (Table 4). This trend for C6 alcohols has been reported by several researchers [5,63,65,70], but only Romero attributed the increase in C6 alcohol concentration to greater ADH activity in ripe fruits based on studies on Chilean VOOs [71]. The results of extensive research indicated that the intensity of fruitiness is closely related to the synthesis of *Z*-3-hexen-1-ol by the action of ADH. On the other hand, the formation of *E*-2-hexen-1-ol, which is preceded by the isomerization of *E*-3-hexenal to *E*-2-hexenal, is favoured in VOOs with medium fruitiness [72]. *Z*-3-hexen-1-ol and especially *E*-2-hexen-1-ol have long been cited as indicators of olive fruit overripeness [73]. In a three-year study, a general increase in hexanol concentration was observed with increasing fruit maturity (R = 0.307). When the studied cultivars were considered separately, a high positive correlation of hexanol concentration with increasing maturity was found, but only for the cultivars Oblica and Lastovka (R = 0.742 and R = 0.525, respectively). Three-year hexanol concentrations ranged from 0.05 ± 0.01 to 1.94 ± 0.16 mg kg^−1^ for Oblica, from 0.02 ± 0.00 to 1.08 ± 0.14 mg kg^−1^ for Levantinka, and from 0.02 to 0.80 ± 0.0 mg kg^−1^ for Lastovka. Two extremely high concentrations of hexanol were detected in 2013 in the VOOs Oblica (3.95 ± 0.18 mg kg^−1^) and Lastovka (5.94 ± 0.29 mg kg^−1^) with high MI. Both values were detected in overripe fruits characterised by the accumulation of free fatty acids (FFAs), which we recorded (Supplementary Materials Table S1a,c). It is known that the LOX enzyme has a 100-fold higher affinity for FFA than for esterified acids [74]. This and the high activity of the LOX enzyme during this period (Figure 1) indicate the formation of substrates that enrich the LOX pathway, which could lead to a higher hexanol content in these oils (Table 2 and Table 4). It has already been mentioned that C6 volatiles depend not only on enzyme activity but also on substrate availability [5]. This is confirmed by the work of Sánchez-Ortiz et al. (2007), who found an increase in the content of desirable C6 volatiles in the Picula and Abequina VOOs by adding linoleic acid and/or linolenic acid to the olive paste during the malaxation process [60]. A high concentration of this alcohol was also found in the Istrian cultivar Buža and the Italian cultivar Pendolino [75]. Other hexanol concentrations in this study are in agreement with the ranges reported for the Spanish and Tunisian cultivars [76]. Although hexanol was not originally considered a good analytical indicator of ripening, it has been considered an indicator of ripening and differences between cultivars for twenty years [21]. In general, we recorded the highest hexanol levels in the oils obtained from the processing of 2/3 of the fruits, which had a purple colour (MI~3.5). These results partially confirmed our expectations. For the Oblica and Lastovka cultivars, we confirmed that the increase in MI increased the activity of the enzyme LOX as well as the concentration of hexanol, which were positively correlated with each other (R = 0.268). Although a positive correlation can be expected for all three cultivars studied, the Levantinka cultivar contradicts this assumption. Since we have the results of a three-year study, it is possible to attribute this pattern of hexanol to a cultivar-specific characteristic that distinguishes Levantinka from Oblica and Lastovka. In the VOOs studied, an almost linear decrease in the concentration of 1-penten-3-ol (Table 2, Table 3 and Table 4) was observed, accompanied by an increase in MI, which is thus strongly negatively correlated with it (R = −0.730). This distribution pattern of 1-penten-3-ol and the concomitant increase in LOX enzyme activity indicate that as maturation progresses, the primary branch of the LOX pathway, which gives rise to the C6 volatile compounds, becomes dominant. The decrease in this alcohol associated with buttery and soft-green odours has also been described in Mission and Paragon VOOs [21]. In the same study, the concentration of 1-penten-3-ol remained the same in Leccino and Corregiola oils, while it increased significantly in Manzanilla oils. These data served to highlight 1-penten-3-ol first as an indicator of cultivar differences and then as another ripening indicator, which was also confirmed by the data obtained in this study, as all three varieties studied showed the same pattern of concentration change during olive fruit ripening (Table 2, Table 3 and Table 4).

In addition to the compounds mentioned so far, it should be mentioned that the concentration of volatile esters in the VOOs of studied cultivars was significantly lower than the concentration of volatile aldehydes and alcohols (Table 2, Table 3 and Table 4). The expected low concentrations of volatile C6 esters can be attributed to the low activity of the alcohol acetyl transferase (ATT) towards short-chain alcohols [77]. As ripening progresses, a gradual increase in hexyl acetate concentration is observed, with a significant correlation between hexyl acetate concentration and MI with R = 0.600 (Table 2, Table 3 and Table 4). When looking at the individual cultivars, significant positive correlations were observed for all three, but it is evident that the strength of the correlation ranges from R = 0.372 for Lastovka to Oblica, which has a significant positive correlation of R = 0.534 to the strongest significant positive correlation between hexyl acetate concentration and MI for Levatinka at R = 0.757. Consistent with the above, we observed a significantly higher value of hexyl acetate (3.94 ± 0.22 mg kg^−1^) in Levatinka oil obtained by processing ripe fruits in 2015 (MI = 5.2) (Table 3). In Oblica (0.69 ± 0.09 mg kg^−1^) and Lastovka (0.26 ± 0.01 mg kg^−1^) oils, we demonstrated an overall positive correlation of R = 0.448 between this C6 volatile ester, associated with the desirable sweet and fruity odour of the VOOs, and LOX enzyme activity in the fruit. By cultivar, the strongest correlation was observed in Levantinka with R = 0.633, followed by Oblica and Lastovka with values of R = 0.536 and R = 0.365, respectively. The highest concentrations of volatile esters were found in the oils obtained by processing riper fruits (Table 2, Table 3 and Table 4). Table S4, containing the listed *p* values for all previous statistical one-way ANOVA analyses, was added to the Supplementary Material.

From the data presented above, it can be concluded that the premature harvesting of olives produces olive oil with less fruitiness (lower concentrations of LA C6 and LAn C6) and more bitterness and pungency (high PC content), i.e., oils that are not harmonious/well balanced. The harmony of the oil is one of the positive characteristics of olive oil [78] (IOC/T.20-Doc.-No-4-Rev1-2007), and it can be seen that the relationship between the positive sensory characteristics (the most important of which are fruitiness, bitterness, pungency) is very close in terms of values. Applying the results of this research in practice could bring us closer to the production of balanced oils.

## 3. Materials and Methods

### 3.1. Plant Material and Olive Oil Extraction

The fruits of *Olea europea* L., three autochthonous Dalmatian cultivars Oblica, Levantinka and Lastovka, collected from the same three trees per cultivar in the orchard cultivated under integrated pest management practice, were harvested in three consecutive years from 2013 to 2015 in the period from October to December, and the interval between harvests was two weeks. In 2014, some parts of the fruits were damaged by *Bactrocera oleae*, so we excluded them from the study. The fruits were picked by hand, and some of the fruits were immersed in liquid nitrogen, stored at −80 °C and used for the study of enzyme activity. Some of the fruits were processed into oil.

### 3.2. Protein Extraction

Protein extraction was performed according to a previously tested protocol [79]. The pulp was weighed and immersed in a buffer to homogenise the composition: 100 mM Na_2_PO_4_ pH 6.8, 1 mM EDTA, 2 g PVPP, 0.1% Triton X-100, 0.1 mM PMFS and 5 mM α-aminocaproic acid. The mixture was kept on ice and homogenised using a Polytron PT 1600 E (Kinematica, Germany). Homogenization was performed in four cycles of 30 s with 1 min pause at 15,000 rpm. The homogenised mixture was filtered through two layers of Miracloth (Millipore, Germany) and centrifuged at 10,000× *g* for 10 min at 4 °C. The concentration of total proteins in the collected supernatants was determined using the Bradford method [80]. Solutions of a protein standard (bovine serum albumin, BSA, Sigma Aldrich, St. Louis, MO, USA) at concentrations from 0 to 1.0 mg mL^−1^ (coefficient of determination R^2^ = 0.9992) were used to generate a calibration curve. The same buffer containing the protein of interest was used to prepare the BSA solution. The protein concentration was determined in microtiter plates in a specially adapted EL808 reader (Bio-Tek Instruments, Winooski, VT, USA). The same volume (5 µL) of the standard or sample was added to 250 µL of the freshly prepared and filtered composition: Coomassie Brilliant Blue G-250 (0.1 g mL^−1^), ethanol (ψ = 5%) and phosphoric acid (ψ = 10%). The colour was developed for 5 min to 1 h before the absorbance was measured at 595 nm. The samples were stored at −20 °C and used to measure LOX activity.

### 3.3. Determination of LOX Activity

#### 3.3.1. Synthesis and Isolation of Hydroperoxide

The synthesis and isolation of linoleic acid hydroperoxide was performed according to a previously published protocol [79]. In brief, the enzyme mixture (2.5 mL) consisted of 0.1 M MES buffer (pH 6.0), protein extract (100 µg mL^−1^) and linoleic acid (250 µm) and was stirred at 27 °C for 30 min. The enzymatic reaction was stopped via the addition of HCl (1 M) to pH 2. The isolation of 9- and 13-*Z*,*E*-HPOD from the reaction mixture was monitored using an internal standard, butylated hydroxytoluene (BHT) (0.22 mmol L^−1^). The products of LOX enzyme activity were separated from the reaction mixture in a series of three extractions using a hexane in 2-propanol mixture (95:5 *v*/*v*). The collected extracts were evaporated in a nitrogen stream and dissolved in 200 to 600 mL of mobile phase acetonitrile in a water mixture (67:33 *v*/*v*).

#### 3.3.2. RP-HPLC Analysis

The products were separated via RP-HPLC (Perkin Elmer Series 200, Waltham, MA, USA), as previously reported [79]. To improve the separation, two Zorbax Eclipse XDB-C18 columns (5 µm, 4.6 mm × 250 mm and 5 µm, 4.6 mm × 150 mm Agilent) connected in series were used and tempered at 35 °C. A binary pump controlled the flow of solvent A (0.25% acetic acid) and solvent B (100% acetonitrile). Gradient elution of the analyte was achieved with a pump program: from 0–22 min, 37% of solvent A flowed; at 22–25 min, the flow of solvent A was reduced to 20% and maintained until 47 min. The flow of solvent A increased to 37% in the period 47–49 min and remained the same until the end of the analysis in 63 min. The volume of the injected sample was 10 μL, while the flow rate of the mobile phase was 0.8 mL min^−1^. The LOX reaction products were detected with a UV/Vis detector at 234 nm and quantified with external standards (±)9-*Z*,*E*-HPOD and (±)13-*Z*,*E*-HPOD (Cayman Chemicals, Ann Arbor, MI, USA).

Three analyses were performed for each sample, and the results were expressed in µg mL^−1^.

### 3.4. Olive Oil Extraction

A laboratory oil mill from Abencor (MC2, Ingenierias y Sistems, Seville, Spain) was used to obtain the oil. Olive fruits (1 kg) were ground using a hammer mill with 3000 rpm and a 5 mm sieve. The resulting paste was mixed at 26 ± 1 °C for 35 min and centrifuged at 3500 rpm for 90 s. The oil was separated from the water by decantation and stored in dark glass bottles filled to the top. The oils were stored in the dark at 4 °C until analysis.

#### 3.4.1. Physicochemical Analysis of Oil

The basic quality parameters of the oils obtained were determined by measuring the physicochemical parameters, namely the free fatty acid content (FFA) expressed as % oleic acid, the peroxide value (PV) expressed as meq O_2_ kg^−1^ and the specific absorption coefficients in the confirmed UV range at 232 and 270 nm (K_232_, K_270_ and ΔK). The analyses were performed according to the analytical methods described in the Commission Regulation (EEC) No 2568/91 and its subsequent amendments [81].

#### 3.4.2. Extraction of Phenolic Compounds

The isolation of phenolic compounds from the hydrophilic fraction of the oil was performed according to the official method of the IOC (IOC/T.20/Doc. No 29/November 2009) [82]. In a screw-capped test tube, 2 g of oil was weighed, and 1 mL of the solvent methanol/water (80:20 *v*/*v*) was added. The contents were mixed with a vortexer for 30 s. Then, 5 mL of methanol/water solvent (80:20 *v*/*v*) was added to the mixture and mixed with a vortexer for 1 min. The prepared mixture was placed in an ultrasonic bath (Bandelin electronic, Berlin) for 15 min at room temperature. The contents were then centrifuged at 5000× *g* for 25 min. The supernatant, the hydrophilic fraction, was collected and filtered through a 0.45 µm cellulose acetate filter. Three parallel isolations were performed, and samples were stored at −20 °C until analysis.

#### 3.4.3. Determination of Total Phenolic Compounds

The content of total phenolic compounds was determined spectrophotometrically via the modified Folin–Ciocalteu method [83]. Although the method used is the usual choice for the determination of TPC concentration, it is possible that the presence of interfering non-phenolic substances leads to increased apparent concentrations of phenolics; therefore, we can conclude that this method measures the reducing capacity of the sample and not only the content of phenolic compounds in the extract.

The colourimetric reaction was performed in a 25 mL volumetric flask to which 250 µL of the isolated hydrophilic fraction, 15 mL of distilled water, and 1.25 mL of Folin–Ciocalteu reagent (Sigma-Aldrich, St. Louis, MO, USA) was added. After 3 min, 2.5 mL of saturated sodium carbonate solution was added, and the flask was filled up to the mark with distilled water. After incubation in the dark for 90 min with occasional stirring at room temperature, the absorbance was measured at 725 nm using a Perkin-Elmer Lambda Bio 40 UV/VIS spectrophotometer versus the blank. The concentration of total phenolic compounds was calculated from an external standard for which caffeic acid was used in the concentration range of 0.03 to 1.0 mg mL^−1^ (R^2^ = 0.9998). The results are expressed as mg of caffeic acid per kg of oil.

#### 3.4.4. Extraction and Analysis of Volatile Compounds

The isolation of volatiles from the oil was performed via solid-phase headspace microextraction (HS-SPME). Then, 3 g of oil was weighed into a 10 mL vial and immediately sealed with a silicone stopper. The contents were thermostated in a water bath at 40 °C for 15 min. Steam extraction on the solid phase was performed at 40 °C for 30 min, followed by thermal desorption of the extracted compounds in the injector of the gas chromatograph at a temperature of 250 °C for 10 min. Analysis of the volatiles in the VOO samples was performed using a Varian GC 3900 gas chromatograph (Varian, Palo Alto, CA, USA) equipped with a split/split injector, a flame ionization detector and a coupled CP-WAX 57 quartz column. It had a 50 m long CB, 0.25 mm inner diameter and 0.2 µm liquid phase layer thickness (Varian, USA). The mobile phase was as follows: helium with a flow rate of 2.5 mL min^−1^ and a pressure of 22.6 psi. A temperature program was used with an initial temperature of 40 °C, which was increased to 200 °C at a rate of 5 °C/4 min, and an isothermal final phase of 10 min. The flame ionization detector (FID) was maintained at 300 °C. The volatile compounds were identified by comparison with the retention time of the standards. The calibration lines for the standards used showed a linear range with a correlation coefficient between 0.966 and 0.999. The concentration ranges of the volatile substances with the corresponding correlation coefficients for the individual standards used were: hexanal (0.08–5.53 mg kg^−1^, R^2^ = 0.992), 1-hexanol (0.03–11.06 mg kg^−1^, R^2^ = 0.985), hexyl acetate (0.03–31.62 mg kg^−1^, R^2^ = 0.991), (*E*)-2-hexenal (0.04–42.27 mg kg^−1^, R^2^ = 0.992), (*E*)-2-hexen-1-ol (0.06–30.39 mg kg^−1^, R^2^ = 0.985), (*Z*)-3-hexen-1-ol (0.05–30.09 mg kg^−1^, R^2^ = 0.981), pentanal (0.09–10.43 mg kg^−1^, R^2^ = 0.998) and 1-penten-3-ol (0.10–21.69 mg kg^−1^, R^2^ = 0.966). Three consecutive analyses of volatile compounds were performed for each oil sample. Data were analysed using the GC Workstation 6.41 chromatographic software tool (Varian, USA).

### 3.5. Statistical Analysis

The experiment was set up as a completely random design in three replications. A one-way analysis of variance (ANOVA) was performed for all the data, separately for each cultivar and study year, with harvest time as the main factor. Comparisons of means were based on Tukey’s post hoc test at *p* ≤ 0.05. To determine the relationship between the analysed parameters, a Pearson correlation coefficient analysis was performed. The correlation was considered significant at a value of *p* ≤ 0.05, and only significant correlations are included in the results and discussion section. The strength of the association was denoted as follows: the absolute values of R are 0–0.19 as very weak, 0.2–0.39 as weak, 0.40–0.59 as moderate, 0.6–0.79 as strong and 0.8–1 as very strong correlation. The analysis was performed using Statistica 14.0.0.15 (Tibco Software Inc, Palo Alto, CA, USA, 2020).

## 4. Conclusions

Lipoxygenase activity varied among cultivars. Despite the differences among them, we found that ripening had a very similar effect on the LOX enzyme activity of the cultivars Oblica, Levantinka and Lastovka. With ripening, the specific activity of LOX enzyme in fruits increases and reaches the highest activity in the period of fruit discolouration (MI~4), followed by a decrease in PC content by more than 75% in Oblica, 60% in Levantinka, and 70% in Lastovka VOO. Analysis of the primary products of linoleic acid oxidation revealed that the LOX enzyme synthesises a mixture of hydroperoxides, namely 9- and 13-*Z*,*E*-HPOD in a ratio of about 1:2 in the cultivars studied, which classifies it as a nontraditional lipoxygenase from the LOX-1 superfamily. The dual specificity isoform of the LOX enzyme has a high affinity for linoleic acid and synthesises 13-*Z*,*E*-HPOD, a precursor of the C6 volatile targets hexanal, hexanol and hexyl acetate. These compounds justified the named maturity indicators during the three years of research on the studied cultivars, as we observed an increase in their concentration as ripening progressed (5.68 mg kg^−1^ in Oblica, 4.33 mg kg^−1^, Levantinka and 7.23 mg kg^−1^ in Lastovka). In the Oblica and Lastovka cultivars, it was confirmed that the increase in MI increased the activity of the LOX enzyme as well as the concentration of hexanol in the VOOs extracted therefrom, while no positive correlation was found in the Levantinka cultivar, although a tendency towards an increase in hexanol with ripening was observed. A positive correlation between MI, LOX enzyme activity and hexyl acetate concentration was observed in all three studied cultivars. The highest concentration of the target components isolated in this study, as well as most of the other desirable C6 volatile compounds (41.16 mg kg^−1^ in Oblica, 40.23 mg kg^−1^ in Levantinka and 34.06 mg kg^−1^ in Lastovka), was found in VOOs obtained by processing fruits with 2/3 purple skin (MI~3). Quantitative differences were found in the concentrations of individual aldehydes, alcohols and esters. The VOOs of the Lastovka cultivar contained significantly lower concentrations of volatiles and lower LOX enzyme activity than those of the Oblica and Levantinka cultivars. The results of this study help to determine the optimal harvest time for the Oblica, Levantinka and Lastovka cultivars and understand the relationships between endogenous enzyme activity and the characteristics of the final product. The results of this study help to understand the development of volatiles according to the degree of ripeness of the fruit in order to achieve harmony with respect to the oil. Based on this idea, it is necessary to include possible factors that influence the activity of LOX enzymes, as well as other enzymes of the LOX pathway, in subsequent tests, with the aim of obtaining practical implications for other olive varieties in the world.

## Figures and Tables

**Table 1:** The table shows the values of the fruit maturity index (MI) for the cultivars Oblica, Levantinka and Lastovka in the period from 2013 to 2015.

Harvest Time	1	2	3	4	5	6
Ob-13	0.23	0.45	1.17	2.07	3.69	4.15
Ob-14	0.73	1.21	2.41	3.83	4.98	-
Ob-15	0.62	1.14	3.06	4.17	4.36	-
Le-13	0.53	0.91	1.91	2.99	3.71	4.05
Le-14	0.43	1.56	2.74	3.91	4.28	-
Le-15	0.68	1.97	2.11	4.49	5.17	5.88
La-13	0.70	1.18	1.80	2.21	2.53	3.23
La-14	0.90	1.37	2.37	2.79	3.32	-
La-15	0.79	1.19	2.45	3.11	3.50	3.64

Identification: Ob: Oblica; Le: Levantinka; La: Lastovka; the harvest year is indicated by the last two numbers (13—2013; 14—2014; and 15—2015).

**Table 2:** The table shows the concentrations of volatile compounds (mg kg^−1^) in virgin olive oils of the Oblica cultivar as a function of harvest time and harvest year.

Volatile Compounds (mg kg^−1^)														
	Ob-13							Ob-14		Ob-15				
	1	2	3	4	5	6	1	2	3	1	2	3	4	5
HEX	1.60 ± 0.09 ^bc^	1.53 ± 0.05 ^cd^	2.07 ± 0.18 ^b^	2.84 ± 0.15 ^a^	1.80 ± 0.14 ^bc^	1.03 ± 0.17 ^d^	2.10 ± 0.18 ^b^	2.99 ± 0.23 ^b^	4.49 ± 0.24 ^a^	1.00 ± 0.09 ^b^	1.12 ± 0.08 ^b^	1.68 ± 0.14 ^a^	1.09 ± 0.08 ^b^	0.43 ± 0.05 ^c^
1Hol	n.d.	0.10 ± 0.00 ^c^	0.10 ± 0.00 ^c^	0.57 ± 0.01 ^b^	0.85 ± 0.06 ^b^	3.96 ± 0.18 ^a^	0.06 ± 0.00 ^c^	0.27 ± 0.01 ^b^	0.50 ± 0.04 ^a^	0.05 ± 0.01 ^d^	0.08 ± 0.01 ^d^	0.67 ± 0.02 ^c^	1.33 ± 0.04 ^b^	1.94 ± 0.16 ^a^
HAC	n.d.	n.d.	n.d.	n.d.	0.30 ± 0.02 ^b^	0.69 ± 0.09 ^a^	n.d.	n.d.	0.05 ± 0.00	0.03 ± 0.00 ^d^	n.d.	0.13 ± 0.00 ^a^	0.10 ± 0.00 ^b^	0.05 ± 0.00 ^c^
LA C6	1.60 ± 0.09 ^d^	1.63 ± 0.05 ^d^	2.18 ± 0.17 ^cd^	3.41 ± 0.14 ^b^	2.95 ± 0.18 ^bc^	5.68 ± 0.43 ^a^	2.16. ± 0.18 ^c^	3.26 ± 0.22 ^b^	5.04 ± 0.24 ^a^	1.08 ± 0.08 ^b^	1.20 ± 0.09 ^b^	2.48 ± 0.15 ^a^	2.52 ± 0.12 ^a^	2.42 ± 0.21 ^a^
E2H	14.06 ± 0.41 ^c^	14.49 ± 0.03 ^c^	23.45 ± 1.51 ^b^	32.61 ± 2.43 ^a^	23.51 ± 1.20 ^b^	2.49 ± 0.11 ^d^	24.19 ± 0.84 ^c^	31.38 ± 1.44 ^b^	40.72 ± 1.19 ^a^	6.39 ± 0.28 ^d^	9.99 ± 0.54 ^c^	27.88 ± 1.19 ^a^	13.01 ± 0.69 ^b^	8.69 ± 0.22 ^cd^
E2Hol	0.27 ± 0.01 ^c^	0.45 ± 0.06 ^c^	0.29 ± 0.01 ^c^	0.30 ± 0.02 ^c^	3.86 ± 0.20 ^b^	5.67 ± 0.15 ^a^	0.04 ± 0.00 ^c^	0.81 ± 0.01 ^b^	1.16 ± 0.03 ^a^	0.46 ± 0.04 ^b^	0.89 ± 0.08 ^a^	0.70 ± 0.07 ^a^	0.19 ± 0.02 ^d^	0.31 ± 0.03 ^bc^
*Z*3Hol	0.67 ± 0.02 ^e^	0.77 ± 0.07 ^e^	1.56 ± 0.07 ^d^	3.99 ± 0.16 ^b^	3.41 ± 0.25 ^c^	4.82 ± 0.12 ^a^	0.82 ± 0.01 ^c^	1.57 ± 0.02 ^b^	3.16 ± 0.13 ^a^	1.09 ± 0.08 ^d^	1.11 ± 0.06 ^d^	5.50 ± 0.42 ^b^	2.53 ± 0.14 ^c^	7.66 ± 0.38 ^a^
LnA C6	15.00 ± 0.44 ^d^	15.71 ± 0.10 ^d^	25.29 ± 1.42 ^c^	36.91 ± 1.73 ^a^	30.78 ± 1.21 ^b^	12.98 ± 0.38 ^d^	25.05 ± 0.35 ^c^	33.77 ± 1.44 ^b^	45.04 ± 1.09 ^a^	7.95 ± 0.40 ^d^	11.99 ± 0.68 ^c^	33.95 ± 1.54 ^a^	15.72 ± 1.09 ^b^	16.66 ± 0.18 ^b^
Pen	0.15 ± 0.01 ^c^	0.13 ± 0.01 ^c^	0.17 ± 0.00 ^c^	0.21 ± 0.00 ^c^	0.97 ± 0.01 ^b^	2.71 ± 0.08 ^a^	0.47 ± 0.02 ^c^	0.70 ± 0.02 ^a^	0.58 ± 0.02 ^b^	0.64 ± 0.07 ^a^	0.25 ± 0.00 ^c^	0.28 ± 0.00 ^c^	0.32 ± 0.02 ^bc^	0.43 ± 0.01 ^b^
Pen3ol	1.10 ± 0.06 ^a^	1.23 ± 0.03 ^a^	0.63 ± 0.02 ^b^	0.63 ± 0.02 ^b^	0.33 ± 0.03 ^c^	0.55 ± 0.03 ^b^	1.06 ± 0.08 ^a^	0.87 ± 0.01 ^a^	0.63 ± 0.03 ^b^	2.20 ± 0.06 ^a^	0.97 ± 0.07 ^b^	0.46 ± 0.01 ^c^	0.47 ± 0.02 ^c^	0.44 ± 0.02 ^c^
C5	1.25 ± 0.07 ^b^	1.36 ± 0.04 ^b^	0.79 ± 0.01 ^c^	0.84 ± 0.02 ^c^	1.30 ± 0.04 ^b^	3.26 ± 0.11 ^a^	1.53 ± 0.07 ^a^	1.56 ± 0.3 ^a^	1.20 ± 0.00 ^b^	2.84 ± 0.10 ^a^	1.22 ± 0.07 ^b^	0.74 ± 0.01 ^c^	0.79 ± 0.04 ^c^	0.87 ± 0.03 ^c^
TVC	17.85 ± 0.60 ^d^	18.69 ± 0.19 ^d^	28.27 ± 1.61 ^c^	41.16 ± 2.17 ^a^	35.06 ± 1.39 ^b^	21.91 ± 0.93 ^d^	28.73 ± 0.71 ^c^	38.59 ± 1.63 ^b^	51.29 ± 0.85 ^a^	11.87 ± 0.30 ^c^	14.41 ± 0.84 ^c^	37.18 ± 1.69 ^a^	19.02 ± 1.00 ^b^	19.95 ± 0.42 ^b^

Identification: Ob: Oblica; year of study is represented by the last two numbers (13—2013; 14—2014 and 15—2015), harvest time (1–6); HEX: hexanal; 1Hol: 1-hexanol; HAC: hexyl acetate; LA C6: the sum of the concentrations of C6 volatile compounds preceding linoleic acid (hexanal, 1-hexanol, and hexyl acetate); E2H: *(E*)-2-hexenal; E2Hol: (*E*)-2-hexen-1-ol; Z3Hol: (*Z*)-3-hexen-1-ol; LnA C6: the sum of the concentrations of C6 volatile compounds preceded by linoleic acid ((*E*)-2-hexenal, (*E*)-2-hexen-1-ol, and (*Z*)-3-hexen-1-ol); Pen: pentanal; Pen3ol: 1-penten-3-ol; C5: the sum of pentanal and 1-penten-3-ol; TVC: the total sum of volatile compound; n.d.: not detected. The results represent the mean of three determinations ± SD. Different superscript lowercase letters represent statistically significant differences between volatile compound mean values at different harvest times (for the specific study year) obtained via a one-way ANOVA and Tukey’s post hoc test (at *p* ≤ 0.05).

**Table 3:** The table shows the concentrations of volatile compounds (mg kg^−1^) in virgin olive oils of the Levantinka cultivar as a function of harvest time and harvest year.

Volatile Compounds (mg kg^−1^)															
			Le-13					Le-14					Le-15		
	1	2	3	4	5	6	1	2	3	1	2	3	4	5	6
HEX	1.16 ± 0.09 ^bc^	1.39 ± 0.07 ^ab^	1.43 ± 0.13 ^ab^	1.65 ± 0.11 ^a^	1.29 ± 0.11 ^abc^	0.89 ± 0.10 ^c^	1.78 ± 0.17 ^a^	3.63 ± 0.31 ^a^	3.05 ± 0.25 ^a^	0.90 ± 0.04 ^a^	0.80 ± 0.08 ^a^	0.58 ± 0.04 ^b^	0.37 ± 0.02 ^c^	0.18 ± 0.01 ^d^	0.09 ± 0.01 ^d^
1Hol	0.02 ± 0.00 ^d^	0.49 ± 0.03 ^b^	0.25 ± 0.02 ^cd^	0.26 ± 0.01 ^bc^	0.35 ± 0.02 ^bc^	1.08± 0.14 ^a^	0.03 ± 0.00 ^b^	0.09 ± 0.00 ^a^	0.08 ± 0.01 ^a^	0.15 ± 0.02 ^c^	0.12 ± 0.01 ^c^	0.26 ± 0.02 ^b^	0.45 ± 0.02 ^a^	0.21 ± 0.00 ^b^	0.46 ± 0.01 ^a^
HAC	n.d.	n.d.	0.19 ± 0.01 ^c^	0.59 ± 0.02 ^a^	0.51 ± 0.02 ^b^	0.07 ± 0.00 ^d^	0.05 ± 0.00 ^b^	n.d.	0.60 ± 0.01 ^a^	0.28 ± 0.03 ^e^	0.59 ± 0.03 ^de^	1.79 ± 0.08 ^c^	0.80 ± 0.02 ^d^	3.94 ± 0.22 ^a^	3.37 ± 0.15 ^b^
LA C6	1.17 ± 0.09 ^c^	1.88 ± 0.04 ^b^	1.87 ± 0.13 ^b^	2.50 ± 0.11 ^a^	2.15 ± 0.08 ^ab^	2.04 ± 0.24 ^ab^	1.85 ± 0.17 ^b^	3.72 ± 0.31 ^a^	3.72 ± 0.24 ^a^	1.33 ± 0.01 ^c^	1.51 ± 0.12 ^c^	2.63 ± 0.12 ^b^	1.62 ± 0.07 ^c^	4.33 ± 0.21 ^a^	3.92 ± 0.17 ^a^
E2H	19.16 ± 1.08 ^c^	22.96 ± 1.38 ^c^	23.64 ± 1.01 ^bc^	33.84 ± 1.69 ^a^	29.91 ± 2.74 ^ab^	11.87 ± 1.14 ^d^	30.20 ± 1.09 ^b^	47.29 ± 2.06 ^a^	43.58 ± 1.71 ^a^	26.28 ± 1.35 ^b^	28.97 ± 2.15 ^ab^	34.23 ± 1.78 ^a^	25.16 ± 1.37 ^b^	25.98 ± 1.60 ^b^	25.50 ± 1.17 ^b^
E2Hol	0.29 ± 0.01 ^d^	1.70 ± 0.11 ^bc^	0.65 ± 0.03 ^d^	1.26 ± 0.07 ^c^	1.90 ± 0.14 ^b^	4.41 ± 0.27 ^a^	0.17 ± 0.02 ^b^	0.36 ± 0.01 ^a^	0.27 ± 0.04 ^ab^	1.33 ± 0.11 ^a^	0.77 ± 0.02 ^bc^	0.80 ± 0.08 ^bc^	0.61 ± 0.07 ^cd^	0.43 ± 0.02 ^d^	0.98 ± 0.02 ^b^
*Z*3Hol	0.47 ± 0.07 ^c^	3.26 ± 0.19 ^a^	1.61 ± 0.09 ^b^	1.65 ± 0.08 ^b^	1.94 ± 0.16 ^b^	0.80 ± 0.04 ^c^	0.41 ± 0.10 ^b^	0.96 ± 0.06 ^a^	0.83 ± 0.05 ^a^	2.50 ± 0.18 ^ab^	1.10 ± 0.12 ^d^	1.66 ± 0.03 ^c^	2.06 ± 0.13 ^bc^	0.85 ± 0.06 ^d^	2.54 ± 0.06 ^a^
LnA C6	19.92 ± 1.03 ^be^	27.92 ± 1.08 ^bc^	25.90 ± 1.13 ^cd^	36.75 ± 1.68 ^a^	33.75 ± 3.04 ^ab^	17.07± 1.45 ^e^	30.78 ± 1.12 ^b^	48.61 ± 2.01 ^a^	44.68 ± 1.73 ^a^	30.10 ± 0.23 ^b^	30.84 ± 2.19 ^ab^	36.69 ± 1.89 ^a^	27.83 ± 1.43 ^b^	27.26 ± 1.56 ^b^	29.02 ± 0.26 ^b^
Pen	0.15 ± 0.01 ^d^	0.87 ± 0.04 ^a^	0.17 ± 0.01 ^d^	0.22 ± 0.02 ^d^	0.38 ± 0.03 ^c^	0.64 ± 0.03 ^b^	0.23 ± 0.03 ^a^	0.20 ± 0.01 ^ab^	0.14 ± 0.01 ^b^	0.39 ± 0.02 ^a^	0.16 ± 0.00 ^cd^	0.23 ± 0.00 ^b^	0.15 ± 0.01 ^cd^	0.12 ± 0.01 ^d^	0.17 ± 0.01 ^c^
Pen3ol	1.13 ± 0.04 ^a^	1.14 ± 0.03 ^a^	0.55 ± 0.02 ^c^	0.72 ± 0.04 ^b^	0.32 ± 0.02 ^d^	0.30 ± 0.02 ^d^	1.20 ± 0.07 ^a^	0.70 ± 0.05 ^b^	0.66 ± 0.02 ^b^	1.69 ± 0.15 ^a^	0.76 ± 0.05 ^b^	0.68 ± 0.01 ^bc^	0.46 ± 0.01 ^cd^	0.34 ± 0.01 ^d^	0.27 ± 0.00 ^d^
C5	1.28 ± 0.05 ^b^	2.01 ± 0.07 ^a^	0.72 ± 0.02 ^d^	0.93 ± 0.00 ^c^	0.70 ± 0.01 ^d^	0.94 ± 0.05 ^c^	1.43 ± 0.09 ^a^	0.91 ± 0.06 ^b^	0.80 ± 0.04 ^b^	2.07 ± 0.17 ^a^	0.92 ± 0.05 ^b^	0.91 ± 0.01 ^b^	0.60 ± 0.02 ^c^	0.46 ± 0.02 ^c^	0.44 ± 0.01 ^c^
TVC	22.38 ± 1.17 ^de^	31.81 ± 1.11 ^bc^	28.49 ± 1.28 ^cd^	40.17 ± 1.57 ^a^	36.60 ± 3.10 ^ab^	20.05 ± 1.74 ^e^	34.06 ± 0.95 ^b^	53.25 ± 2.32 ^a^	49.20 ± 1.93 ^a^	33.51 ± 1.53 ^b^	33.26 ± 2.16 ^b^	40.23 ± 1.86 ^a^	30.08 ± 1.49 ^b^	32.04 ± 1.75 ^b^	33.38 ± 0.42 ^b^

Identification: Le: Levantinka; year of study is represented by the last two numbers (13—2013; 14—2014 and 15—2015), harvest time (1–6); HEX: hexanal; 1Hol: 1-hexanol; HAC: hexyl acetate; LA C6: the sum of the concentrations of C6 volatile compounds preceding linoleic acid (hexanal, 1-hexanol, and hexyl acetate); E2H: *(E*)-2-hexenal; E2Hol: (*E*)-2-hexen-1-ol; Z3Hol: (*Z*)-3-hexen-1-ol; LnA C6: the sum of the concentrations of C6 volatile compounds preceded by linoleic acid ((*E*)-2-hexenal, (*E*)-2-hexen-1-ol, and (*Z*)-3-hexen-1-ol); Pen: pentanal; Pen3ol: 1-penten-3-ol; C5- the sum of pentanal and 1-penten-3-ol; TVC: total sum of volatile compounds; n.d.: not detected. The results represent the mean of three determinations ± SD. Different superscript lowercase letters represent statistically significant differences between volatile compound mean values at different harvest times (for the specific study year) obtained via a one-way ANOVA and Tukey’s post hoc test (at *p* ≤ 0.05).

**Table 4:** The table shows the concentrations of volatile compounds (mg kg^−1^) in virgin olive oils of the Lastovka cultivar as a function of harvest time and harvest year.

Volatile Compounds (mg kg^−1^)															
				La-13				La-14					La-15		
	1	2	3	4	5	6	1	2	3	1	2	3	4	5	6
HEX	0.81 ± 0.04 ^cd^	1.09 ± 0.06 ^bc^	1.17 ± 0.07 ^b^	1.59 ± 0.12 ^a^	1.26 ± 0.09 ^b^	0.66 ± 0.04 ^d^	1.21 ± 0.08 ^b^	1.87 ± 0.10 ^a^	2.02 ± 0.13 ^a^	0.39 ± 0.02 ^d^	0.46 ± 0.02 ^cd^	0.48 ± 0.03 ^c^	0.58 ± 0.03 ^b^	0.74 ± 0.03 ^a^	0.44 ± 0.02 ^cd^
1Hol	0.04 ± 0.00 ^c^	0.03 ± 0.00 ^c^	0.04 ± 0.00 ^c^	0.14 ± 0.01 ^c^	5.94 ± 0.29 ^a^	0.80 ± 0.04 ^b^	0.02 ± 0.00 ^a^	0.04 ± 0.01 ^b^	0.11 ± 0.00 ^a^	0.07 ± 0.01 ^b^	0.06 ± 0.01 ^b^	0.07 ± 0.00 ^b^	0.09 ± 0.00 ^a^	0.07 ± 0.00 ^b^	0.07 ± 0.00 ^b^
HAC	n.d.	n.d.	n.d.	n.d.	0.03 ± 0.00 ^b^	0.03 ± 0.00 ^a^	0.03 ± 0.00 ^b^	0.03 ± 0.00 ^b^	0.08 ± 0.00 ^a^	0.19 ± 0.01 ^b^	0.17 ± 0.01 ^bc^	0.16 ± 0.00 ^bc^	0.14 ± 0.01 ^c^	0.26 ± 0.01 ^a^	0.14 ± 0.01 ^c^
LA C6	0.85 ± 0.03 ^d^	1.12 ± 0.06 ^cd^	1.21 ± 0.08 ^cd^	1.73 ± 0.12 ^b^	7.23 ± 0.20 ^a^	1.49 ± 0.00 ^bc^	1.26 ± 0.11 ^b^	1.95 ± 0.10 ^a^	2.21 ± 0.13 ^a^	0.65 ± 0.03 ^c^	0.68 ± 0.00 ^c^	0.71 ± 0.02 ^c^	0.81 ± 0.01 ^b^	1.07 ± 0.04 ^a^	0.66 ± 0.03 ^c^
E2H	5.81 ± 0.28 ^d^	13.21 ± 0.72 ^bc^	16.21 ± 0.55 ^b^	27.48 ± 1.25 ^a^	13.17 ± 0.88 ^bc^	10.59 ± 1.07 ^c^	14.95 ± 1.35 ^b^	21.81 ± 0.90 ^a^	21.12 ± 0.71 ^a^	6.31 ± 0.07 ^d^	12.19 ± 0.97 ^c^	13.82 ± 0.54 ^c^	13.65 ± 0.92 ^c^	21.37 ± 1.14 ^b^	31.93 ± 1.33 ^a^
E2Hol	0.24 ± 0.01 ^b^	0.36 ± 0.01 ^b^	0.37 ± 0.02 ^b^	0.58 ± 0.06 ^b^	3.98 ± 0.16 ^a^	4.02 ± 0.30 ^a^	0.09 ± 0.01 ^b^	0.08 ± 0.00 ^b^	0.95 ± 0.03 ^a^	0.32 ± 0.01 ^d^	0.43 ± 0.02 ^c^	0.50 ± 0.03 ^bc^	0.60 ± 0.02 ^a^	0.54 ± 0.03 ^ab^	0.46 ± 0.01 ^bc^
*Z*3Hol	0.48 ± 0.03 ^bc^	0.32 ± 0.01 ^d^	0.34 ± 0.02 ^d^	0.40 ± 0.03 ^cd^	0.83 ± 0.07 ^a^	0.60 ± 0.02 ^b^	0.25 ± 0.00 ^a^	0.18 ± 0.01 ^b^	0.24 ± 0.02 ^a^	0.97 ± 0.03 ^a^	0.78 ± 0.03 ^b^	0.87 ± 0.02 ^ab^	0.86 ± 0.07 ^ab^	0.63 ± 0.04 ^c^	0.34 ± 0.03 ^d^
LnA C6	6.53 ± 0.32 ^d^	13.90 ± 0.72 ^c^	16.91 ± 0.52 ^bc^	28.46 ± 1.35 ^a^	17.98 ± 1.11 ^b^	15.20 ± 1.38 ^bc^	15.29 ± 1.36 ^b^	22.07 ± 0.91 ^a^	22.31 ± 0.76 ^a^	7.60 ± 0.09 ^d^	13,39 ± 0.96 ^c^	15.19 ± 0.58 ^c^	15.11 ± 0.97 ^c^	22.54 ± 1.15 ^b^	32.73 ± 1.34 ^a^
Pen	0.25 ± 0.01 b^c^	0.18 ± 0.01 ^c^	0.13 ± 0.01 ^c^	0.09 ± 0.01	3.16 ± 0.16 ^a^	0.52 ± 0.04 ^b^	0.48 ± 0.03 ^b^	0.58 ± 0.01 ^a^	0.65 ± 0.02 ^a^	0.63 ± 0.03 ^a^	0.49 ± 0.02 ^b^	0.21 ± 0.02 ^c^	0.19 ± 0.02 ^cd^	0.12 ± 0.01 ^de^	0.10 ± 0.01 ^e^
Pen3ol	0.86 ± 0.04 ^a^	0.82 ± 0.03 ^a^	0.76 ± 0.04 ^ab^	0.67 ± 0.02 ^b^	0.35 ± 0.04 ^c^	0.26 ± 0.01 ^c^	1.14 ± 0.11	0.94 ± 0.03	0.94 ± 0.08	1.03 ± 0.07 ^a^	0.98 ± 0.05 ^a^	0.96 ± 0.07 ^a^	0.99 ± 0.03 ^a^	0.93 ± 0.02 ^a^	0.57 ± 0.02 ^b^
C5	1.11 ± 0.03 ^b^	1.00 ± 0.04 ^bc^	0.89 ± 0.03 ^bc^	0.75 ± 0.03 ^c^	3.51 ± 0.20 ^a^	0.78 ± 0.05 ^bc^	1.62 ± 0.14	1.52 ± 0.04	1.59 ± 0.06	1.66 ± 0.04 ^a^	1.47 ± 0.07 ^a^	1.16 ± 0.08 ^b^	1.18 ± 0.05 ^b^	1.05 ± 0.01 ^b^	0.67 ± 0.01 ^c^
TVC	8.50 ± 0.37 ^c^	16.01 ± 0.83 ^b^	19.01 ± 0.47 ^b^	30.94 ± 1.44 ^a^	28.72 ± 1.10 ^a^	17.47 ± 1.42 ^b^	18.16 ± 1.42 ^b^	25.54 ± 0.97 ^a^	26.11 ± 0.69 ^a^	9.91 ± 0.02 ^d^	15.55 ± 0.89 ^c^	17.06 ± 0.68 ^c^	17.10 ± 1.01 ^c^	24.66 ± 1.18 ^b^	34.06 ± 1.37 ^a^

Identification: La: Lastovka; year of study is represented by the last two numbers (13—2013; 14—2014 and 15—2015), harvest time (1–6); HEX: hexanal; 1Hol: 1-hexanol; HAC: hexyl acetate;LA C6: the sum of the concentrations of C6 volatile compounds preceding linoleic acid (hexanal, 1-hexanol and hexyl acetate); E2H: *(E*)-2-hexenal; E2Hol: (*E*)-2-hexen-1-ol; Z3Hol: (*Z*)-3-hexen-1-ol; LnA C6: the sum of the concentrations of C6 volatile compounds preceded by linoleic acid ((*E*)-2-hexenal, (*E*)-2-hexen-1-ol, and (*Z*)-3-hexen-1-ol); Pen: pentanal; Pen3ol: 1-penten-3-ol; C5: the sum of pentanal and 1-penten-3-ol; TVC: total sum of volatile compounds; n.d.: not detected. The results represent the mean of three determinations ± SD. Different superscript lowercase letters represent statistically significant differences between volatile compound mean values at different harvest times (for the specific study year) obtained via a one-way ANOVA and Tukey’s post hoc test (at *p* ≤ 0.05).

## Data Availability

The original contributions presented in the study are included in the article and Supplementary Material; further requests can be addressed to the corresponding author.

## Supplementary Materials

The following supporting information can be downloaded at: https://www.mdpi.com/article/10.3390/molecules29081696/s1. Table S1: Quality parameters of virgin olive oils of the Oblica cultivar; Table S2: Quality parameters of virgin olive oils of the Levantinka cultivar; Table S3: Quality parameters of virgin olive oils of the Lastovka cultivar, Figure S1 Average monthly precipitation (mm) measured for 2013, 2014 and 2015 at the Kaštela meteorological station and Table S4: One-way ANOVA *p*-values for each of the selected variables previously listed for different cultivars and study years in Table 2, Table 3 and Table 4 as well as in Figure 1 and Figure 2.

## References

[1] Correia M., Moreira I., El Maghariki J., Manuel T., Alves P., Barros R., Gomes A. (2023). The Metabolic and Analytical Changes of Healthy Volunteers upon Intake of Portuguese Extra Virgin Olive Oil: A Comparison Study between Pre- and Post-Intervention. Nutrients.

[2] Foscolou A., Critselis E., Panagiotakos D. (2018). Olive Oil Consumption and Human Health: A Narrative Review. Maturitas.

[3] Millman J.F., Okamoto S., Teruya T., Uema T., Ikematsu S., Shimabukuro M., Masuzaki H. (2021). Extra-virgin Olive Oil and the Gut-brain Axis: Influence on Gut Microbiota, Mucosal Immunity, and Cardiometabolic and Cognitive Health. Nutr. Rev..

[4] Cecchi L., Migliorini M., Mulinacci N. (2021). Virgin Olive Oil Volatile Compounds: Composition, Sensory Characteristics, Analytical Approaches, Quality Control, and Authentication. J. Agric. Food Chem..

[5] Kalua C.M., Allen M.S., Bedgood D.R., Bishop A.G., Prenzler P.D., Robards K. (2007). Olive Oil Volatile Compounds, Flavour Development and Quality: A Critical Review. Food Chem..

[6] Angerosa F. (2002). Influence of Volatile Compounds on Virgin Olive Oil Quality Evaluated by Analytical Approaches and Sensor Panels. Eur. J. Lipid Sci. Technol..

[7] Aparicio R., Luna G. (2002). Characterisation of Monovarietal Virgin Olive Oils. Eur. J. Lipid Sci. Technol..

[8] Sanchez J., Salas J., Harwood J., Aparicio R. (2013). Biogenesis of Olive Oil Aroma. Handbook of Olive Oil: Analysis and Properties.

[9] Schiller D., Contreras C., Vogt J., Dunemann F., Defilippi B.G., Beaudry R., Schwab W. (2015). A Dual Positional Specific Lipoxygenase Functions in the Generation of Flavor Compounds During Climacteric Ripening of Apple. Hortic. Res..

[10] Palmieri-Thiers C., Canaan S., Brunini V., Lorenzi V., Tomi F., Desseyn J.L., Garscha U., Oliw E.H., Berti L., Maury J. (2009). A Lipoxygenase With Dual Positional Specificity is Expressed in Olives (*Olea europaea* L.) During Ripening. Biochim. Biophys. Acta BBA—Mol. Cell Biol. Lipids.

[11] Padilla M.N., Hernández M.L., Sanz C., Martínez-Rivas J.M. (2009). Functional Characterization of Two 13-Lipoxygenase Genes from Olive Fruit in Relation to the Biosynthesis of Volatile Compounds of Virgin Olive Oil. J. Agric. Food Chem..

[12] Ridolfi M., Terenziani S., Patumi M., Fontanazza G. (2002). Characterization of the Lipoxygenases in Some Olive Cultivars and Determination of Their Role in Volatile Compounds Formation. J. Agric. Food Chem..

[13] Angerosa F., Servili M., Selvaggini R., Taticchi A., Esposto S., Montedoro G. (2004). Volatile Compounds in Virgin Olive Oil: Occurrence and Their Relationship With the Quality. J. Chromatogr. A.

[14] Rotondi A., Bendini A., Cerretani L., Mari M., Lercker G., Toschi T.G. (2004). Effect of Olive Ripening Degree on the Oxidative Stability and Organoleptic Properties of Cv. Nostrana di Brisighella Extra Virgin Olive Oil. J. Agric. Food Chem..

[15] Şişik Oğraş S., Kaban G., Kaya M. (2018). Volatile Compounds of Olive Oils from Different Geographic Regions in Turkey. Int. J. Food Prop..

[16] Mikrou T., Litsa M., Papantoni A., Kapsokefalou M., Gardeli C., Mallouchos A. (2023). Effect of Cultivar and Geographical Origin on the Volatile Composition of Greek Monovarietal Extra Virgin Olive Oils. Chemosensors.

[17] Lima A.F., Da Silva Oliveira W., De Oliveira Garcia A., Vicente E., Godoy H.T. (2023). Identifying Markers Volatiles in Brazilian Virgin Oil by Multiple Headspace Solid-phase Microextraction, and Chemometrics Tools. Food Res..

[18] Šarolić M., Gugić M., Friganović E., Tuberoso C., Jerković I. (2015). Phytochemicals and Other Characteristics of Croatian Monovarietal Extra Virgin Olive Oils from Oblica, Lastovka and Levantinka Varieties. Molecules.

[19] Popović M., Jukić Špika M., Veršić Bratinčević M., Ninčević T., Matešković A., Mandušić M., Rošin J., Nazlić M., Dunkić V., Vitanović E. (2021). Essential Oil Volatile Fingerprint Differentiates Croatian cv. Oblica from Other *Olea europaea* L. Cultivars. Molecules.

[20] Bendini A., Cerretani L., Carrasco-Pancorbo A., Gómez-Caravaca A.M., Segura-Carretero A., Ferná A. (2007). Phenolic Molecules in Virgin Olive Oils: A Survey of Their Sensory Properties, Health Effects, Antioxidant Activity and Analytical Methods. An Overview of the Last Decade Alessandra. Molecules.

[21] Kalua C.M., Allen M.S., Bedgood D.R., Bishop A.G., Prenzler P.D. (2005). Discrimination of Olive Oils and Fruits into Cultivars and Maturity Stages Based on Phenolic and Volatile Compounds. J. Agric. Food Chem..

[22] Di Lecce G., Piochi M., Pacetti D., Frega N.G., Bartolucci E., Scortichini S., Fiorini D. (2020). Eleven Monovarietal Extra Virgin Olive Oils from Olives Grown and Processed under the Same Conditions: Effect of the Cultivar on the Chemical Composition and Sensory Traits. Foods.

[23] Ramos-Escudero F., Morales M.T., Asuero A.G. (2015). Characterization of Bioactive Compounds from Monovarietal Virgin Olive Oils: Relationship Between Phenolic Compounds-Antioxidant Capacities. Int. J. Food Prop..

[24] Škevin D., Rade D., Štrucelj D., Mokrovšak Ž., Neđeral S., Benčić Đ. (2003). The Influence of Variety and Harvest Time on the Bitterness and Phenolic Compounds of Olive Oil. Eur. J. Lipid Sci. Technol..

[25] Bonoli M., Bendini A., Cerretani L., Lercker G., Gallina Toschi T. (2004). Qualitative and Semiquantitative Analysis of Phenolic Compounds in Extra Virgin Olive Oils as a Function of the Ripening Degree of Olive Fruits by Different Analytical Techniques. J. Agric. Food Chem..

[26] Bengana M., Bakhouche A., Lozano-Sánchez J., Amir Y., Youyou A., Segura-Carretero A., Fernández-Gutiérrez A. (2013). Influence of Olive Ripeness on Chemical Properties and Phenolic Composition of Chemlal Extra Virgin Olive Oil. Food Res. Int..

[27] Uceda M., Frias L. (1975). Harvest Dates. Evolution of the Fruit Oil Content, Oil Composition and Oil Quality. Proceedings of the Del Segundo Seminario Oleicola Internacional.

[28] Sanz C.L., Perez G.A., Rios J.J., Olias M.J. (1993). Positional Specificity of Ketodienes from Linoleic Acid Aerobically Formed by Lipoxygenase Isozymes from Kidney Bean and Pea. J. Agric. Food Chem..

[29] De Gregorio A., Dugo G., Arena N. (2000). Lipoxygenase Activites in Ripening Olive Fruit Tissue. J. Food Biochem..

[30] Salas J.J., Willams M., Harwood J.L., Sánchez J. (1999). Lipoxygenase Activity in Olive (*Olea europaea*) Fruit. J. Am. Oil Chem. Soc..

[31] Palmieri-Thiers P., de Caraffa V.B.B., Lorenzi V., Gambotti C., Giannettini J., Berti L., Maury J. (2009). Biochemical and molecular aspects of olive lipoxygenase. Advances in olive resources.

[32] Luaces P., Sanz C., Pérez A.G. (2007). Thermal Stability of Lipoxygenase and Hydroperoxide Lyase from Olive Fruit and Repercussion on Olive Oil Aroma Biosynthesis. J. Agric. Food Chem..

[33] European Union Commission (2022). Commission Delegated Regulation 2022/2104 of 29 July 2022 supplementing Regulation (EU) No 1308/2013 of the European Parliament and of the Council as regards marketing standards for olive oil, and repealing Commission Regulation (EEC) No 2568/91 Commission Implementing Regulation (EU) No 29/2012. J. Eur. Union.

[34] Žanetić M., Jukić Špika M., Ožić M.M., Brkić Bubola K. (2021). Comparative Study of Volatile Compounds and Sensory Characteristics of Dalmatian Monovarietal Virgin Olive Oils. Plants.

[35] Farinelli D., Tombesi S. (2015). Performance and Oil Quality of ‘Arbequina’and Four Italian Olive Cultivars Under Super High Density Hedgerow Planting System Cultivated in Central Italy. Sci. Hortic..

[36] Jukić Špika M., Perica S., Žanetić M., Škevin D. (2021). Virgin Olive Oil Phenols, Fatty Acid Composition and Sensory Profile: Can Cultivar Overpower Environmental and Ripening Effect?. Antioxidants.

[37] Kalogianni E.P., Georgiou D., Hasanov J.H. (2019). Olive Oil Processing: Current Knowledge, Literature Gaps, and Future Perspectives. J. Am. Oil Chem. Soc..

[38] Genovese A., Caporaso N., Villani V., Paduano A., Sacchi R. (2015). Olive Oil Phenolic Compounds Affect the Release of Aroma Compounds. Food Chem..

[39] Genovese A., Caporaso N., Sacchi R. (2021). Flavor Chemistry of Virgin Olive Oil: An Overview. Appl. Sci..

[40] Rodríguez-López P., Lozano-Sánchez J., Borras-Linares I., Emanuelli T., Menendez J.A., Segura-Carretero A. (2021). Polyphenols in Olive Oil. Olives and Olive Oil in Health and Disease Prevention.

[41] Celano R., Piccinelli A.L., Pugliese A., Carabetta S., Di Sanzo R., Rastrelli L., Russo M. (2018). Insights into the Analysis of Phenolic Secoiridoids in Extra Virgin Olive Oil. J. Agric. Food Chem..

[42] Jukić Špika M., Liber Z., Montemurro C., Miazzi M.M., Ljubenkov I., Soldo B., Žanetić M., Vitanović E., Politeo O., Škevin D. (2022). Quantitatively Unraveling Hierarchy of Factors Impacting Virgin Olive Oil Phenolic Profile and Oxidative Stability. Antioxidants.

[43] Dag A., Ben-Gal A., Yermiyahu U., Basheer L., Nir Y., Kerem Z. (2008). The Effect of Irrigation Level and Harvest Mechanization on Virgin Olive Oil Quality in a Traditional Rain-fed ‘Souri’ Olive Orchard Converted to Irrigation. J. Sci. Food Agric..

[44] Gómez-Rico A., Desamparados Salvador M., Moriana A., Pérez D., Olmedilla N., Ribas F., Fregapane G. (2007). Influence of Different Irrigation Strategies in a Traditional Cornicabra cv. Olive Orchard on Virgin Olive Oil Composition and Quality. Food Chem..

[45] Notario A., Sánchez R., Luaces P., Sanz C., Pérez A.G. (2022). The Infestation of Olive Fruits by Bactrocera oleae (Rossi) Modifies the Expression of Key Genes in the Biosynthesis of Volatile and Phenolic Compounds and Alters the Composition of Virgin Olive Oil. Molecules.

[46] Aguilera M.P., Beltrán G., Ortega D., Fernández A., Jiménez A., Uceda M. (2005). Characterisation of Virgin Olive Oil of Italian Olive Cultivars: `Frantoio’ and `Leccino’, Grown in Andalusia. Food Chem..

[47] Conte P., Caponio G., Difonzo G., Fadd C., Del Caro A., Urgeghe P.P., Montanari L., Montinaro A., Piga A. (2019). Change in Quality During Ripening of Olive Fruits and Related Oils Extracted from Three Minor Autochthonous Sardinian Cultivars. Emir. J. Food Agric..

[48] Deiana P., Molinu M.G., Dore A., Culeddu N., Dettori S., Santona M. (2023). Evolution of Monovarietal Virgin Olive Oils as a Function of Chemical Composition and Oxidation Status. Nat. Prod. Res..

[49] Vidal A.M., Alcalá S., De Torres A., Moya M., Espínola F. (2019). Characterization of Olive Oils from Superintensive Crops with Different Ripening Degree, Irrigation Management, and Cultivar: (Arbequina, Koroneiki, and Arbosana). Eur. J. Lipid Sci. Technol..

[50] López-Yerena A., Ninot A., Jiménez-Rui N., Lozano-Castelló J., Pérez M., Escribano-Ferrer E., Romero-Aroca A., Lamuela-Raventós R.M., Vallverdú-Queralt A. (2021). Influence of the Ripening Stage and Extraction Conditions on the Phenolic Fingerprint of ‘Corbella’ Extra-Virgin Olive Oil. Antioxidants.

[51] De Torres A., Espínola F., Moya M., Alcalá S., Vidal A.M., Castro E. (2018). Assessment of Phenolic Compounds in Virgin Olive Oil by response Surface Methodology With Particular Focus on Flavonoids and Lignans. LWT.

[52] Romero-Segura C., García-Rodríguez R., Sánchez-Ortiz A., Sanz C., Pérez A.G. (2012). The Role of Olive β-glucosidase in Shaping the Phenolic Profile of Virgin Olive Oil. Food Res. Int..

[53] Loomis W.D., Battaile J. (1966). Plant Phenolic Compounds and the Isolation of Plant Enzymes. Phytochemistry.

[54] Muzzalupo I., Stefanizzi F., Perri E., Chiappetta A. (2012). Variation of the Antioxidant Compounds in Italian Olive (*Olea europea*) Drupes During Ripening Stage. Acta Hortic..

[55] Miho H., Moral J., López-González M.A., Díez C.M., Priego-Capote F. (2020). The Phenolic Profile of Virgin Olive Oil is Influenced by Malaxation Conditions and Determines the Oxidative Stability. Food Chem..

[56] Germek V.M., Koprivnjak O., Butinar B., Pizzale L., Bučar-Miklavčič M., Conte L.S. (2013). Influence of Phenols Mass Fraction in Olive (*Olea europaea* L.) Paste on Volatile Compounds in Buža Cultivar Virgin Olive Oil. J. Agric. Food Chem..

[57] Brkić Bubola K., Koprivnjak O., Sladonja B., Lukić I. (2012). Volatile Compounds and Sensory Profiles of Monovarietal Virgin Olive Oil from Buža, Črna and Rosinjola Cultivars in Istria (Croatia). Food Technol. Biotechnol..

[58] Kotti F., Cerretani L., Gargouri M., Chiavaro E., Bendini A. (2011). Evaluation of the Volatile Fraction of Commercial Virgin Olive Oils From Tunisia and Italy: Relation With Olfactory Attributes. J. Food Biochem..

[59] Haddada F.M., Manai H., Daoud D., Fernandez X., Lizzani-Cuvelier L., Zarrouk M. (2007). Profiles of Volatile Compounds From Some Monovarietal Tunisian Virgin Olive Oils. Comparison with French PDO. Food Chem..

[60] Sánchez-Ortiz A., Pérez A.G., Sanz C. (2007). Cultivar Differences on Nonesterified Polyunsaturated Fatty Acid as a Limiting Factor for the Biogenesis of Virgin Olive Oil Aroma. J. Agric. Food Chem..

[61] Rodrigues J.F., Resende L.M.B., de Oliveira da Silva L.F., Pozzobon Pedroso M., Pinheiro A.C.M., Nunes C.A. (2019). Quality of Olive Oils from Southeastern Brazil. Bragantia.

[62] Angerosa F., Basti C., Vito R. (1999). Virgin Olive Oil Volatile Compounds from Lipoxygenase Pathway and Characterization of Some Italian Cultivars. J. Agric. Food Chem..

[63] Baccouri O., Bendini A., Cerretani L., Guerfel M., Baccouri B., Lercker G., Zarrouk M., Ben Miled D.D. (2008). Comparative Study on Volatile Compounds from Tunisian and Sicilian Monovarietal Virgin Olive Oils. Food Chem..

[64] Cevik S., Ozkan G., Kiralan M., Bayrak A. (2014). Effect of Harvest Time on Physicochemical Quality Parameters, Oxidation Stability, and Volatile Compounds of Extra Virgin Olive Oil. Acta Aliment..

[65] Lukić I., Žanetić M., Jukić Špika M., Lukić M., Koprivnjak O., Brkić Bubola K. (2017). Complex Interactive Effects of Ripening Degree, Malaxation Duration and Temperature on Oblica cv. Virgin Olive Oil Phenols, Volatiles and Sensory Quality. Food Chem..

[66] Brkić Bubola K., Koprivnjak O., Sladonja B., Škevin D., Belobrajić I. (2012). Chemical and Sensorial Changes of Croatian Monovarietal Olive Oils During Ripening. Eur. J. Lipid Sci. Technol..

[67] Hachicha Hbaieb R., Kotti F., Vichi S., Gargouri M. (2017). Evolution of Endogenous Enzyme Activities and Virgin Olive Oil Characteristics During Chétoui and Chemlali Olive Ripening. Eur. J. Lipid Sci. Technol..

[68] Salas J. (2000). Simultaneous Determination of the Lipoxygenase and Hydroperoxide lyase Specificity in Olive Fruit Pulp. Grasas Aceites.

[69] Muzzalupo I., Macchione B., Bucci C., Stefanizzi F., Perri E., Chiappetta A., Tagarelli A., Sindona G. (2012). LOX Gene Transcript Accumulation in Olive (*Olea europaea* L.) Fruits at Different Stages of Maturation: Relationship between Volatile Compounds, Environmental Factors, and Technological Treatments for Oil Extraction. Sci. World J..

[70] Gómez-Rico A., Fregapane G., Salvador M.D. (2008). Effect of Cultivar and Ripening on Minor Components in Spanish Olive Fruits and Their Corresponding Virgin Olive Oils. Food Res. Int..

[71] Romero N., Saavedra J., Tapia F., Sepúlveda B., Aparicio R. (2016). Influence of Agroclimatic Parameters on Phenolic and Volatile Compounds of Chilean Virgin Olive Oils and Characterization Based on Geographical Origin, Cultivar and Ripening Stage. J. Sci. Food Agric..

[72] Tomé-Rodríguez S., Ledesma-Escobar C.A., Penco-Valenzuela J.M., Calderón-Santiago M., Priego-Capote F. (2022). Metabolic Patterns in the Lipoxygenase Pathway Associated to Fruitiness Attributes of Extra Virgin Olive Oil. J. Food Compos. Anal..

[73] Aparicio R., Morales M.T. (1998). Characterization of Olive Ripeness by Green Aroma Compounds of Virgin Olive Oil. J. Agric. Food Chem..

[74] Clodoveo M.L., Hbaieb R.H., Kotti F., Mugnozza G.S., Gargouri M. (2014). Mechanical Strategies to Increase Nutritional and Sensory Quality of Virgin Olive Oil by Modulating the Endogenous Enzyme Activities. Compr. Rev. Food Sci. Food Saf..

[75] Runcio A., Sorgonà L., Mincione A., Santacaterina S., Poiana M. (2008). Volatile Compounds of Virgin Olive Oil Obtained from Italian Cultivars Grown in Calabria. Food Chem..

[76] Tena N., Lazzez A., Aparicio-Ruiz R., García-González D.L. (2007). Volatile Compounds Characterizing Tunisian Chemlali and Chétoui Virgin Olive Oils. J. Agric. Food Chem..

[77] Salas J.J. (2004). Characterization of Alcohol Acyltransferase from Olive Fruit. J. Agric. Food Chem..

[78] International Olive Council (2007). Sensory Analysis of Olive Oil, Standard Sensory Analysis of Olive Oil.

[79] Soldo B., Šprung M., Mušac G., Pavela-Vrančić M., Ljubenkov I. (2016). Evaluation of Olive Fruit Lipoxygenase Extraction Protocols on 9- and 13-*Z*,*E*-HPODE Formation. Molecules.

[80] Bradford M. (1976). A Rapid and Sensitive Metod for the Quantitation of Microgram Quantities of Proteines of Porotein Utilising Principle of Rapid-dye Binging. Anal. Biochem..

[81] European Union Commission (1991). Characteristics of Olive Oil and Olive-Residue Oil and the Relevant Methods of Analysis. Regulation EEC/2568/91 and Later Modifications. Off. J. Eur. Community.

[82] International Olive Council (2009). Determination of Biophenols in Olive Oils by HPLC.

[83] Gutfinger T. (1981). Polyphenols in Olive Oils. J. Am. Oil Chem. Soc..

