# Supplementary material for: The Composition of Volatiles and the Role of Non-Traditional LOX on Target Metabolites in Virgin Olive Oil from Autochthonous Dalmatian Cultivars
Source: Molecules. 2024 Apr 9;29(8):1696. doi: 10.3390/molecules29081696 (PMC11051971; doi:10.3390/molecules29081696)
Supplement: Supplementary file 1 [file molecules-29-01696-s001.zip › molecules-2927755-supplementary.docx]

The Composition of Volatiles and the Role of Non-Traditional LOX on Target Metabolites in Virgin Olive Oil from
Autochthonous Dalmatian Cultivars

Barbara Soldo ^1^, Maja Jukić Špika ^2,3^, Igor Pasković ^4,5^, Elma Vuko ^6^, Marija Polić Pasković ^4^ and Ivica Ljubenkov ^1,^*

^1^ Department of Chemistry, Faculty of Science, University of Split, Ruđera Boškovića 33, 21000 Split, Croatia; barbara@pmfst.hr

^2^ Department of Applied Sciences, Institute for Adriatic Crops and Karst Reclamation, Put Duilova 11,
21000 Split, Croatia; maja@krs.hr

^3^ Centre of Excellence for Biodiversity and Molecular Plant Breeding, Svetošimunska 25,
10000 Zagreb, Croatia

^4^ Department of Agriculture and Nutrition, Institute of Agriculture and Tourism, K. Huguesa 8,
52440 Poreč, Croatia; paskovic@iptpo.hr (I.P.); mpolic@iptpo.hr (M.P.P.)

^5^ Faculty of Health Studies, University of Rijeka, Viktora Cara Emina 5, HR-51000 Rijeka, Croatia

^6^ Department of Biology, Faculty of Science, University of Split, Ruđera Boškovića 33, 21000 Split, Croatia; elma@pmfst.hr

***** Correspondence: iljubenk@pmfst.hr; Tel.: +358-21-619-218

**SUPPLEMENTARY MATERIAL**

Table S1. Quality parameters of virgin olive oils of the Oblica cultivar

|  | Harvest time  (HT) | FFA  (% oleic acid) | PV  (meq O_2_ kg^-1^) | K_232_ | K_270_ | ΔK |
| --- | --- | --- | --- | --- | --- | --- |
| Ob-13 | 1 | 0.26 ± 0.01 | 7.26 ± 0.02 | 1.95 ± 0.07 | 0.15 ± 0.01 | -0.01 |
|  | 2 | 0.19 ± 0.01 | 6.20 ± 0.04 | 1.72 ± 0.01 | 0.16 ± 0.04 | -0.01 |
|  | 3 | 0.23 ± 0.01 | 4.96 ± 0.08 | 1.51 ± 0.01 | 0.10 ± 0.01 | 0 |
|  | 4 | 0.24 ± 0.01 | 8.68 ± 0.07 | 1.58 ± 0.08 | 0.13 ± 0.01 | 0 |
|  | 5 | 0.33 ± 0.02 | 6.40 ± 0.02 | 2.03 ± 0.02 | 0.11 ± 0.01 | 0 |
|  | 6 | 1.51 ± 0.08 | 12.64 ± 0.08 | 1.77 ± 0.10 | 0.15 ± 0.02 | 0 |
| Ob-14 | 1 | 0.24 ± 0.01 | 12.20 ± 0.14 | 2.15 ± 0.08 | 0.15 ± 0.01 | 0 |
|  | 2 | 0.29 ± 0.01 | 16.02 ± 0.08 | 2.00 ± 0.09 | 0.13 ± 0.03 | 0 |
|  | 3 | 0.63 ± 0.01 | 14.22 ± 0.28 | 2.05 ± 0.04 | 0.13 ± 0.02 | 0 |
| Ob-15 | 1 | 0.19 ± 0.01 | 4.44 ± 0.08 | 1.90 ± 0.02 | 0.16 ± 0.02 | 0 |
|  | 2 | 0.21 ± 0.02 | 3.94 ± 0.12 | 1.63 ± 0.07 | 0.15 ± 0.02 | 0 |
|  | 3 | 0.31 ± 0.01 | 8.26 ± 0.12 | 1.85 ± 0.01 | 0.12 ± 0.01 | 0.01 |
|  | 4 | 0.47 ± 0.02 | 10.82 ± 0.28 | 2.15 ± 0.02 | 0.12 ± 0.01 | 0.01 |
|  | 5 | 0.65 ± 0.04 | 13.38 ± 0.34 | 1.75 ± 0.05 | 0.17 ± 0.01 | 0 |

Identification; Ob-Oblica; year of study is represented by the last two numbers (13-2013; 14-2014 i 15-2015), HT- harvest time (1-6); FFA- free fatty acid, PV-peroxide value, UV spectrophotometric indices (K_232_ and K_270_, ∆K). Results are expressed as mean values of three repetitions ± standard deviation.

Table S2. Quality parameters of virgin olive oils of the Levantinka cultivar

|  | Harvest time  (HT) | FFA  (% oleic acid) | PV  (meq O_2_ kg^-1^) | K_232_ | K_270_ | ΔK |
| --- | --- | --- | --- | --- | --- | --- |
| Le-13 | 1 | 0.34 ± 0.01 | 6.36 ± 0.16 | 1.92 ± 0.02 | 0.23 ± 0.02 | 0 |
|  | 2 | 0.23 ± 0.01 | 7.74 ± 0.24 | 2.06 ± 0.01 | 0.21 ± 0.06 | 0 |
|  | 3 | 0.22 ± 0.01 | 4.64 ± 0.08 | 1.67 ± 0.05 | 0.12 ± 0.01 | 0 |
|  | 4 | 0.33 ± 0.03 | 5.82 ± 0.20 | 1.64 ± 0.07 | 0.10 ± 0.02 | 0 |
|  | 5 | 0.30 ± 0.02 | 4.28 ± 0.08 | 1.96 ± 0.01 | 0.09 ± 0.01 | 0.01 |
|  | 6 | 0.27 ± 0.01 | 8.14 ± 0.08 | 2.06 ± 0.02 | 0.12 ± 0.01 | 0 |
| Le-14 | 1 | 0.16 ± 0.01 | 12.46 ± 0.04 | 1.64 ± 0.03 | 0.17 ± 0.02 | 0 |
|  | 2 | 0.25 ± 0.01 | 14.72 ± 0.06 | 1.67 ± 0.02 | 0.13 ± 0.01 | 0 |
|  | 3 | 0.25 ± 0.01 | 12.16 ± 0.18 | 1.77 ± 0.04 | 0.14 ± 0.02 | 0 |
| Le-15 | 1 | 0.15 ± 0.01 | 4.22 ± 0.02 | 1.77 ± 0.03 | 0.19 ± 0.02 | 0 |
|  | 2 | 0.18 ± 0.01 | 5.84 ± 0.08 | 1.63± 0.03 | 0.21 ± 0.05 | 0 |
|  | 3 | 0.24 ± 0.03 | 6.86 ± 0.06 | 2.04 ± 0.07 | 0.14 ± 0.01 | 0 |
|  | 4 | 0.38 ± 0.03 | 5.96 ± 0.16 | 2.13± 0.03 | 0.12 ± 0.01 | -0.01 |
|  | 5 | 0.26 ± 0.01 | 9.01 ± 0.07 | 2.19 ± 0.03 | 0.09 ± 0.01 | -0.01 |
|  | 6 | 0.40 ± 0.02 | 8.62 ± 0.12 | 2.02 ± 0.04 | 0.17 ± 0.02 | 0 |

Identification; Le-Levantinka; year of study is represented by the last two numbers (13-2013; 14-2014 i 15-2015), HT- harvest time (1-6); FFA- free fatty acid, PV-peroxide value, UV spectrophotometric indices (K_232_ and K_270_, ∆K). Results are expressed as mean values of three repetitions ± standard deviation.

Table S3. Quality parameters of virgin olive oils of the Lastovka cultivar

|  | Harvest time  (HT) | FFA  (% oleic acid) | PV  (meq O_2_ kg^-1^) | K_232_ | K_270_ | ΔK |
| --- | --- | --- | --- | --- | --- | --- |
| La-13 | 1 | 0.19 ± 0.01 | 7.12 ± 0.24 | 1.73 ± 0.01 | 0.15 ± 0.02 | 0 |
|  | 2 | 0.18 ± 0.01 | 7.76 ± 0.12 | 1.88 ± 0.07 | 0.13 ± 0.01 | 0 |
|  | 3 | 0.19 ± 0.01 | 4.18 ± 0.10 | 1.49 ± 0.09 | 0.16 ± 0.02 | 0 |
|  | 4 | 0.21 ± 0.02 | 8.70 ± 0.24 | 2.00 ± 0.05 | 0.09 ± 0.01 | 0 |
|  | 5 | 0.30 ± 0.03 | 9.18 ± 0.66 | 2.01 ± 0.01 | 0.13 ± 0.01 | 0 |
|  | 6 | 0.31 ± 0.01 | 7.07 ± 0.12 | 1.82 ± 0.05 | 0.12 ± 0.01 | 0 |
| La-14 | 1 | 0.23 ± 0.01 | 5.06 ± 0.08 | 1.69 ± 0.03 | 0.18 ± 0.01 | 0 |
|  | 2 | 0.30 ± 0.01 | 5.28 ± 0.12 | 1.66 ± 0.01 | 0.13 ± 0.03 | 0 |
|  | 3 | 0.82 ± 0.02 | 5.68 ± 0.08 | 1.48 ± 0.12 | 0.13 ± 0.03 | 0 |
| La-15 | 1 | 0.19 ± 0.02 | 6.20 ± 0.04 | 1.88 ± 0.01 | 0.16 ± 0.02 | -0,01 |
|  | 2 | 0.22 ± 0.01 | 6.48 ± 0.02 | 1.97 ± 0.01 | 0.15 ± 0.01 | 0 |
|  | 3 | 0.20 ± 0.01 | 4.42 ± 0.22 | 1.85 ± 0.06 | 0.16 ± 0.01 | 0 |
|  | 4 | 0.32 ± 0.03 | 3.84 ± 0.08 | 2.00 ± 0.02 | 0.12 ± 0.01 | -0,01 |
|  | 5 | 0.36 ± 0.01 | 4.02 ± 0.24 | 2.08 ± 0.07 | 0.15 ± 0.02 | 0 |
|  | 6 | 0.32 ± 0.03 | 3.54 ± 0.16 | 1.87 ± 0.01 | 0.12 ± 0.01 | 0 |

Identification; La-Lastovka; year of study is represented by the last two numbers (13-2013; 14-2014 i 15-2015), HT- harvest time (1-6); FFA- free fatty acid, PV-peroxide value, UV spectrophotometric indices (K_232_ and K_270_, ∆K). Results are expressed as mean values of three repetitions ± standard deviation.


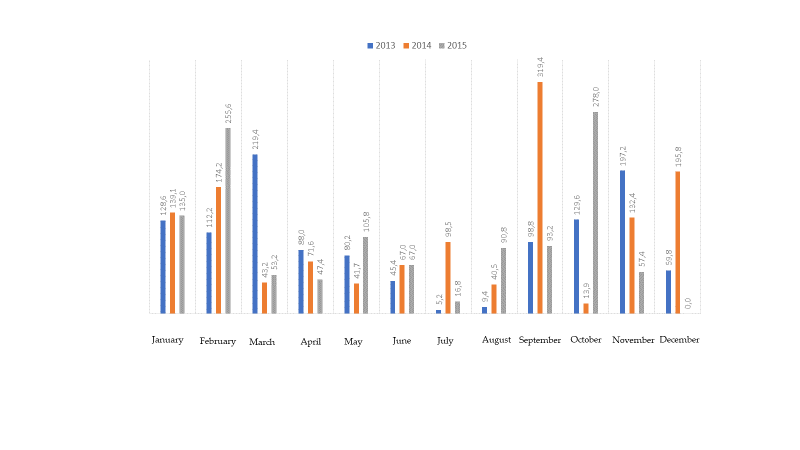


Figure S1. Average monthly precipitation (mm) measured for 2013, 2014 and 2015 at the Kaštela meteorological station. The data was provided by the State Hydrometrological Institute of the Republic of Croatia.

Table S4. One-way ANOVA p-values for each of the selected variables previously listed for different cultivars and study years in Tables 2, 3 and 4 as well as in Figures 1 and 2

| Variable/Cultivar&year | | Ob-13 | Ob-14 | Ob-15 | Le-13 | Le-14 | Le-15 | La-13 | La-14 | La-15 |
| --- | --- | --- | --- | --- | --- | --- | --- | --- | --- | --- |
| heksanal | | <0.001 | 0.004 | <0.001 | 0.005 | 0.011 | <0.001 | <0.001 | 0.009 | <0.001 |
| heksanol | <0.001 | | <0.001 | <0.001 | <0.001 | <0.001 | <0.001 | <0.001 | <0.001 | 0.004 |
| heksil acetat | <0.001 | | <0.001 | <0.001 | <0.001 | <0.001 | <0.001 | <0.001 | <0.001 | <0.001 |
| LA C6 | <0.001 | | 0.002 | <0.001 | <0.001 | 0.007 | <0.001 | <0.001 | 0.006 | <0.001 |
| *E*-2-heksanal | <0.001 | | 0.002 | <0.001 | <0.001 | 0.004 | 0.007 | <0.001 | 0.012 | <0.001 |
| *E*-2-heksen-1-ol | <0.001 | | <0.001 | <0.001 | <0.001 | 0.012 | <0.001 | <0.001 | <0.001 | <0.001 |
| *Z*-3-heksen-1-ol | <0.001 | | <0.001 | <0.001 | <0.001 | 0.008 | <0.001 | <0.001 | 0.01 | <0.001 |
| LnA C6 | <0.001 | | <0.001 | <0.001 | <0.001 | 0.004 | 0.01 | <0.001 | 0.011 | <0.001 |
| pentanal | <0.001 | | 0.003 | <0.001 | <0.001 | 0.032 | <0.001 | <0.001 | 0.009 | <0.001 |
| 1-penten-3-ol | <0.001 | | 0.007 | <0.001 | <0.001 | 0.002 | <0.001 | <0.001 | **0.125** | <0.001 |
| C5 | <0.001 | | <0.001 | <0.001 | <0.001 | 0.003 | <0.001 | <0.001 | **0.595** | <0.001 |
| TVC | <0.001 | | <0.001 | <0.001 | <0.001 | 0.004 | 0.01 | <0.001 | 0.009 | <0.001 |
| TPC | <0.001 | | <0.001 | <0.001 | <0.001 | <0.001 | <0.001 | <0.001 | <0.001 | <0.001 |
| 13-HPOD | <0.001 | | <0.001 | <0.001 | <0.001 | <0.001 | <0.001 | <0.001 | <0.001 | <0.001 |
| 9-HPOD | <0.001 | | <0.001 | <0.001 | <0.001 | <0.001 | <0.001 | <0.001 | <0.001 | <0.001 |

Identification: Ob- Oblica, Le- Levantinka, La- Lastovka; year of study is represented by the last two numbers (13-2013; 14-2014 i 15-2015)
